# Vitamin D supplementation for the treatment of COVID-19: A systematic review and meta-analysis of randomized controlled trials

**DOI:** 10.3389/fimmu.2022.1023903

**Published:** 2022-10-31

**Authors:** Lara S. Kümmel, Hanna Krumbein, Paraskevi C. Fragkou, Ben L. Hünerbein, Rieke Reiter, Konstantinos A. Papathanasiou, Clemens Thölken, Scott T. Weiss, Harald Renz, Chrysanthi Skevaki

**Affiliations:** ^1^ Institute of Laboratory Medicine, Universities of Giessen and Marburg Lung Center (UKGMLC), Philipps Universität Marburg, German Center for Lung Research/ Deutsches Zentrum für Lungenforschung (DZL) Marburg, Marburg, Germany; ^2^ First Department of Critical Care Medicine and Pulmonary Services, Evangelismos Hospital, Medical School of Athens, National and Kapodistrian University of Athens, Athens, Greece; ^3^ Medical School, National and Kapodistrian University of Athens, Athens, Greece; ^4^ Institute of Medical Bioinformatics and Biostatistics, Medical Faculty, Philipps University of Marburg, Marburg, Germany; ^5^ Channing Division of Network Medicine, Department of Medicine, Brigham and Women’s Hospital, Harvard Medical School, Boston, MA, United States

**Keywords:** vitamin D, SARS-CoV-2, COVID-19, systematic review, meta-analysis

## Abstract

Vitamin D supplementation and its impact on immunoregulation are widely investigated. We aimed to assess the prevention and treatment efficiency of vitamin D supplementation in the context of coronavirus disease 2019 (COVID-19) and any disease-related complications. For this systematic review and meta-analysis, we searched databases (PubMed, Embase, Scopus, Web of Science, The Cochrane Library, medRxiv, Cochrane COVID-19 Study Register, and ClinicalTrial.gov) for studies published between 1 November 2019 and 17 September 2021. We considered randomized trials (RCTs) as potentially eligible when patients were tested for SARS-CoV-2 infection and received vitamin D supplementation versus a placebo or standard-of-care control. A random-effects model was implemented to obtain pooled odds ratios for the effect of vitamin D supplementation on the main outcome of mortality as well as clinical outcomes. We identified a total of 5,733 articles, of which eight RCTs (657 patients) met the eligibility criteria. Although no statistically significant effects were reached, the use of vitamin D supplementation showed a trend for reduced mortality [odds ratio (OR) 0.74, 95% confidence interval (CI) 0.32–1.71, *p* = 0.48] compared with the control group, with even stronger effects, when vitamin D was administered repeatedly (OR 0.33, 95% CI 0.1–1.14). The mean difference for the length of hospitalization was −0.28 (95% CI −0.60 to 0.04), and the ORs were 0.41 (95% CI 0.15–1.12) and 0.52 (95% CI 0.27–1.02) for ICU admission and mechanical ventilation, respectively. In conclusion, vitamin D supplementation did not improve the clinical outcomes in COVID-19 patients, but trends of beneficial effects were observed. Further investigations are required, especially studies focusing on the daily administration of vitamin D.

## Introduction

Within a short period of time, the novel coronavirus [severe acute respiratory syndrome-related coronavirus 2 (SARS-CoV-2)] has become a global challenge. The increase in the number of coronavirus disease 2019 (COVID-19) cases dictates the need for low-cost and widely available therapies, to help prevent SARS-COV-2 infections and protect from severe COVID-19.

There is evidence that vitamin D has an important impact on the human immune system and can prevent respiratory tract infections (1), as vitamin D plays a signaling role in the modulation of the innate and adaptive arms of the immune system and immunoregulation. A link has been made between pathogen recognition, cytokine secretion, the expression of nuclear vitamin D receptors, and 1-α-hydroxylase (CYP27B1), which is expressed in several tissues and immune cells (2). The active forms of vitamin D induce the production of antimicrobial peptides and support the differentiation of monocytes, with the enhancement of phagocytic and chemotactic capacity (3). Vitamin D leads to a more tolerogenic immune environment by downregulating the production of proinflammatory cytokines (e.g., IL-12, IFN-γ, IL-6, IL-8, TNF-α, and IL-9) and increasing the anti-inflammatory responses through blocking the NK-κB pathways (e.g., IL-4, IL-5, and IL-10) (4). The indirect and direct effects on T-cell differentiation lead toward a Th2 phenotype, and B-cell proliferation and immunoglobulin secretion are inhibited by vitamin D (3, 5).

The global prevalence of vitamin D deficiency (<20 ng/ml) is high and even higher for partial deficiency (<30 ng/ml). Several studies have reported data on the prevalence of low vitamin D levels in Europe (up to 40%) and in the United States of America, Canada, and India with more than 20% of the general population being deficient, which shows that inadequate vitamin D serum concentrations are a frequent issue around the globe (6). Vitamin D may come from three different sources: endogenous production after exposure to UVB rays, nutritional sources, or exogenous supplementation. Vitamin D has a good safety profile and is associated with a low risk for acute intoxication with commonly recommended doses (2). Therefore, it is a widely available low-cost supplement with the potential to reduce the prevalence of vitamin D deficiency. Recent observational studies have reported heterogeneous results about the association between insufficient vitamin D serum levels and the risk of developing severe COVID-19, requiring further investigations (7).

The aim of this review was to assess the potential effects of vitamin D supplementation on the treatment and prevention of COVID-19 and severity-related complications. In addition, it also aimed to evaluate the impact of different dosing and administration regimens compared with placebo or standard of care in randomized controlled trials (RCTs).

## Materials and methods

### Search strategy and selection criteria

This systematic review and meta-analysis was conducted according to the Preferred Reporting Items for Systematic Reviews and Meta-Analyses (PRISMA) guidelines (**Supplementary Table 6**) (8) and followed the “Cochrane Handbook for Systematic Reviews of Interventions” (9). The study protocol was registered with PROSPERO (CRD42021279150).

Two reviewers (LK, HK) independently performed the systematic search of the databases PubMed/MEDLINE, EMBASE, Scopus, Web of Science, The Cochrane Library, and the preprint server medRxiv in addition to the trial registries Cochrane COVID-19 Study Register and ClinicalTrials.gov for clinical trials published between 1 November 2019 and 17 September 2021. This search was performed using the following search terms: “COVID-19 OR SARS-CoV-2” AND “vitamin D.” The search strategies are available in **Supplementary Table 1**. Additionally, a manual search was performed to identify further records by screening gray literature and references of eligible studies.

Studies were included in this meta-analysis when meeting the following inclusion criteria: involving participants with no age, gender, or ethnicity restriction, who were tested for SARS-CoV-2 infections as defined by the World Health Organization (10); investigating any type of vitamin D supplementation compared with placebo, standard of care, or no treatment; and giving information of relevant clinical outcomes. Furthermore, only RCTs published in English or German language were eligible. We excluded all other types of studies, studies which administered additional or different agents than vitamin D, and studies that did not test for SARS-CoV-2 infections or with missing assessment of the relevant outcomes.

Two independent teams of two reviewers (LK, RR; HK, BH) screened titles and abstracts to identify potentially eligible studies. The same two teams independently screened the full texts of possibly relevant studies. Any disagreements were resolved by consulting an independent fifth reviewer (KP).

### Data analysis

The relevant data of all included studies were extracted and independently reviewed by two reviewers (LK, HK). Data were entered into a predefined table for analysis. The criteria for inclusion were strictly adhered to and any discrepancies were resolved by discussion until an agreement was reached. The corresponding authors of the trials were contacted for important missing data. The risk of bias of the included studies was independently assessed by two reviewers (LK, HK) using the Cochrane Risk of Bias tool for RCTs (11). The tool included five domains, which were rated from low to high risk, and then combined to indicate the overall risk of bias. Any disagreements were resolved by consensus.

Statistical analysis was carried out using the meta (v5.0-0) package (12) in the R (v4.1.2) programming language (13). Meta-analysis of proportions was pooled by fitting a random intercept logistic regression model with the metaprop function to logit-transformed proportions in order to include valid estimates for studies with very few or no events. Study estimates are shown with computed Clopper–Pearson 95% confidence intervals. The same pooled estimates were conducted for subgroups and tested by the *χ* ^2^ test for significant pairwise differences. Heterogeneity was assessed by estimating the maximum likelihood of *τ* ^2^ and quantified with the *I* ^2^ index. Comparisons of studies were analyzed using odds ratios between the treatment and control groups with the metabin function by performing a random-effects model for the pooled odds ratio using the Mantel–Haenszel method (14). Heterogeneity was assessed using a restricted maximum-likelihood estimator of *τ* ^2^.

Publication bias was evaluated by performing a funnel plot of the logit-transformed prevalence and inverse variance. This was tested using the metabias function with the linear regression test (15) and the rank correlation test (16) for asymmetry. Correlations with vitamin D levels were investigated by linear regression of mean concentration per study, weighted by study size (reflected by circle size and opacity). Squared Pearson correlation coefficient (*R* ^2^) is stated per correlation.

The quality of evidence was analyzed by performing the GRADE approach to evaluate the certainty of evidence. Using the GRADE.pro software (17), we created a summary of findings table.

## Results

### Study selection

**Figure 1:**
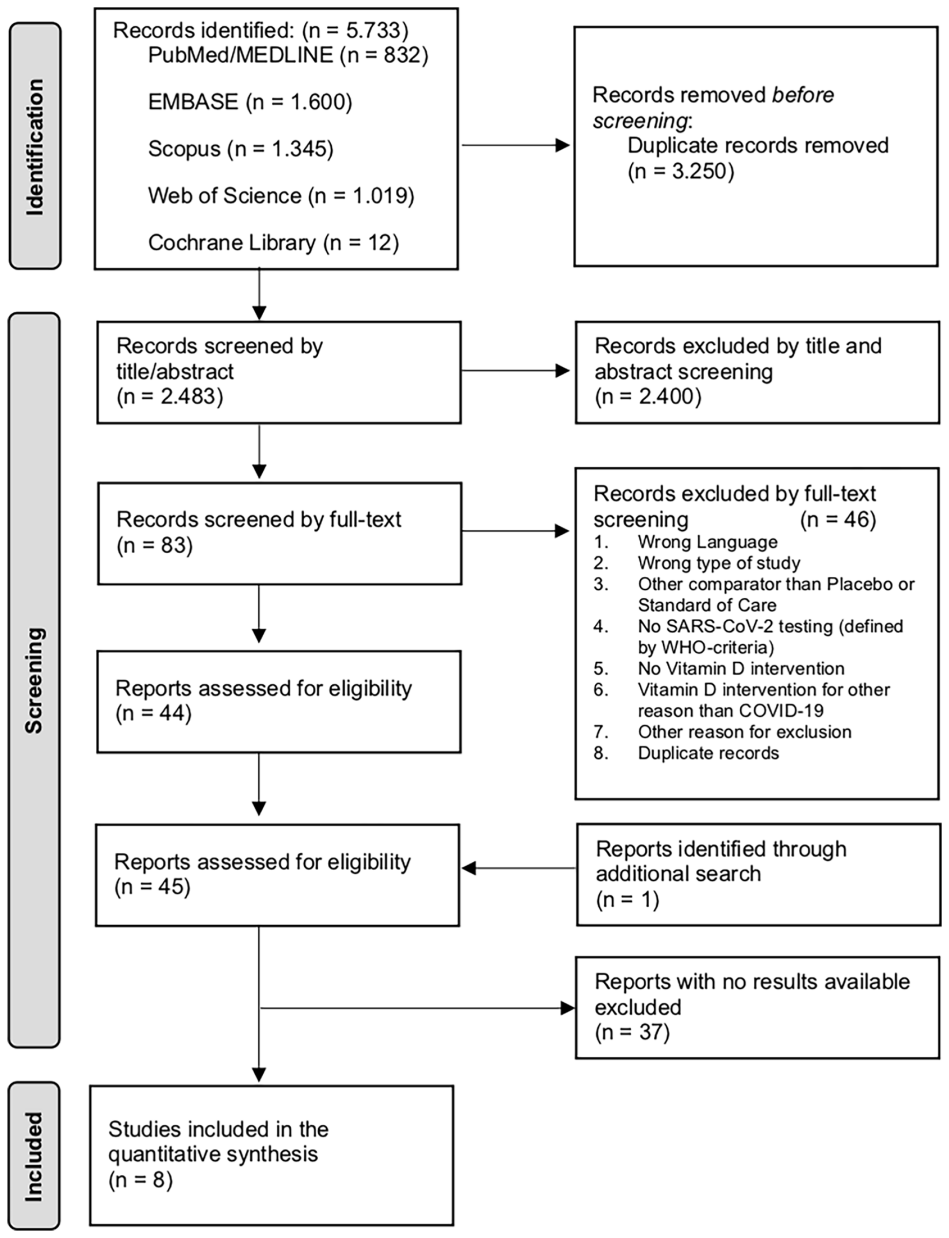
PRISMA flowchart showing the study selection process to identify trials on vitamin D supplementation for the treatment and prevention of COVID-19. COVID-19, coronavirus disease 2019; SARS-CoV-2, severe acute respiratory syndrome-related coronavirus 2; WHO, World Health Organization.

The initial search identified a total of 5,733 articles of potential relevance. After the removal of duplicates, 2,483 articles were screened by title and abstract, and 83 potentially eligible records were selected for full-text reading. In total, 44 RCTs were eligible according to the inclusion criteria; an additional trial was identified by manual search. However, no results were available for 37 of the trials, which were excluded as they did not meet the inclusion criteria. Thus, we were able to finally include eight RCTs in this meta-analysis with a combined total of 657 individual patients (**Figure 1**).

### Study characteristics

All trials included adults of both genders (**Supplementary Figure 1**) (18–25). Two studies were conducted in India, while the remaining six originated from different countries around the world; six studies assessed the data of hospitalized patients and one only included outpatients. The trial by Sabico et al. included both settings. Patients were only included if SARS-CoV-2 infection was confirmed by PCR or other criteria matching the WHO definition. Participants with a vitamin D deficiency were enrolled in three studies; the Murai et al. trial performed an additional *post-hoc* analysis for patients with vitamin D deficiency at baseline. The COVID-19 severity ranged from asymptomatic to severe, although most studies did not report the severity of disease.

Furthermore, three studies compared the effects of vitamin D supplementation with placebo and five with standard of care. The majority of studies used cholecalciferol. However, the dosage and duration of the vitamin D supplementation varied, ranging from 0.5 to 5,000 µg; vitamin D was administered as a single bolus, a daily dose, or using a combination by administrating a high-dose bolus followed by daily doses (trial by Castillo et al.). Relevant data were reported, with mortality in all eight studies, but other outcomes were assessed more infrequently. The timing of follow-up assessments varied across studies, although it was not always specified. Serum vitamin D levels at baseline were measured in five studies only during follow-up, while the others simply reported the baseline serum levels. The baseline characteristics of the participants were heterogeneous, even reporting significant differences of vitamin D serum levels between the intervention and the control groups, when vitamin D concentrations were assessed. Additional characteristics of the included studies are presented in **Table 1**. No prevention trials were included. The overall risk of bias within the studies was assessed and considered to be low to some concerns (**Supplementary Figure 2**).

**Table 1:** Characteristics of the eligible randomized controlled trials and their patients (**18**–**25**).

	Trial design	Participants	Vitamin D deficiency	Groups		Vitamin D treatment	Timing of follow-up	Outcomes (relevant for this meta-analysis)
				Intervention	Control			
Castillo et al., 2020 (Spain)	RCT Open label Single center	Inpatients >18 years Total *N* = 76 Female: 31 Mean age: 53 years	No	Calcifediol *N* = 50 Female: 23 Mean age: 53.14 years	Standard of care *N* = 26 Female: 8 Mean age: 52.77 years	Loading dose of 532 µg, followed by 266 µg on days 3, 7, 14, 21, and 28	Until ICU admission, discharge, or death	Mortality Need for ICU admission
Elamir et al., 2021 (Israel)	RCT Open label Multicenter	Inpatients >18 years Total *N* = 50 Female: 25 Mean age: NR	No	Calcitriol *N* = 25 Female: 13 Mean age: 69 years	Standard of care *N* = 25 Female: 12 Mean age: 64 years	Daily dose of 0.5 µg for 14 days or until discharge	Until day 14 or discharge	Mortality Length of hospitalization Need for ICU admission Need for mechanical ventilation
Lakkireddy et al., 2021 (India)	RCT Open label Single center	Inpatients >18 years Total *\N* = 87 Female: 22 Mean age: 45 years	Yes, defined as <30 ng/ml	Cholecalciferol *N* = 44 Female: 7 Mean age: 47 years Mean vitamin D level: 16 ng/ml (baseline); 89 ng/ml (follow-up)	Standard of care *N* = 43 Female: 15 Mean age: 44 years Mean vitamin D level: 17 ng/ml (baseline); 16 ng/ml (follow-up)	Daily dose of 1,500 µg for 8 or 10 days	Until day 21	Mortality Length of hospitalization Need for ICU admission
Murai et al., 2021 (Brazil)	RCT Double-blind Multicenter	Inpatients >18 years Total *N* = 237 Female: 104 Mean age: 56.2 years	Subgroup, defined as <20 ng/ml	Cholecalciferol *N* = 119 Female: 49 Mean age: 56.5 years Mean vitamin D level: 21 ng/ml (baseline); 44 ng/ml (follow-up)	Placebo *N* = 118 Female: 55 Mean age: 56 years Mean vitamin D level: 20 ng/ml (baseline); 19 ng/ml (follow-up)	Single dose of 5,000 µg	Until discharge	Mortality Length of hospitalization Need for ICU admission Need for mechanical ventilation
Rastogi et al., 2020 (India)	RCT Double-blind Single center	Inpatients >18 years Total *N* = 40 Female: 20 Median age: NR	Yes, defined as <20 ng/ml	Cholecalciferol *N* = 16 Female: 10 Median age: 50 years Median vitamin D level: 8 ng/ml (baseline); 51 ng/ml (follow-up)	Placebo *N* = 24 Female: 10 Median age: 47.5 years Median vitamin D level: 9 ng/ml (baseline); 15 ng/ml (follow-up)	Daily dose of 1,500 µg for 7 or 14 days	Until day 21	Mortality
Sabico et al., 2021 (Saudi Arabia)	RCT Open label Multicenter	In- and outpatients from 20 to 75 years Total *N* = 69 Female: 35 Mean age: 49.8 years	No	Cholecalciferol *N* = 36 Female: 15 Mean age: 46.3 years Mean vitamin D level: 21 ng/ml (baseline); 25 ng/ml (follow-up)	Standard of care, including 25 µg cholecalciferol *N* = 33 Female: 20 Mean age: 53.5 years Mean vitamin D level: 25 ng/ml (baseline); 23 ng/ml (follow-up)	Daily dose of 125 µg for 14 days	Until discharge	Mortality Length of hospitalization Need for ICU admission
Sanchez-Zuno et al., 2021 (Mexico)	RCT Open label Multicenter	Outpatients >18 years Total *N* = 42 Female: 22 Median age: 43 years	No	Cholecalciferol *N* = 22 Female: 7 Median age: 44 years Median vitamin D level: 20 ng/ml (baseline); 28 ng/ml (follow-up)	Standard of care *N* = 20 Female: 6 Median age: 43 years Median vitamin D level: 23 ng/ml (baseline)	Daily dose of 250 µg for 14 days	Until day 14	Mortality
Soliman et al., 2021 (Egypt)	RCT Double-blind Single center	Inpatients >60 years with DM II Total *N* = 56 Female: 22 Mean age: 70.91 years	Yes, defined as <20 ng/ml	Cholecalciferol *N* = 40 Female: 16 Mean age: 71.3 years Mean vitamin D level: 10 ng/ml (baseline); 20 ng/ml (follow-up)	Placebo *N* = 16 Female: 6 Mean age: 70.19 years Mean vitamin D level: 21 ng/ml (baseline); 21 ng/ml (follow-up)	Single dose of 5,000 µg	Until day 42	Mortality Need for mechanical ventilation

DM, diabetes mellitus; N, number of participants; NR, not reported; RCT, randomized controlled trial.

### Results of the meta-analyses

**Figure 2:**
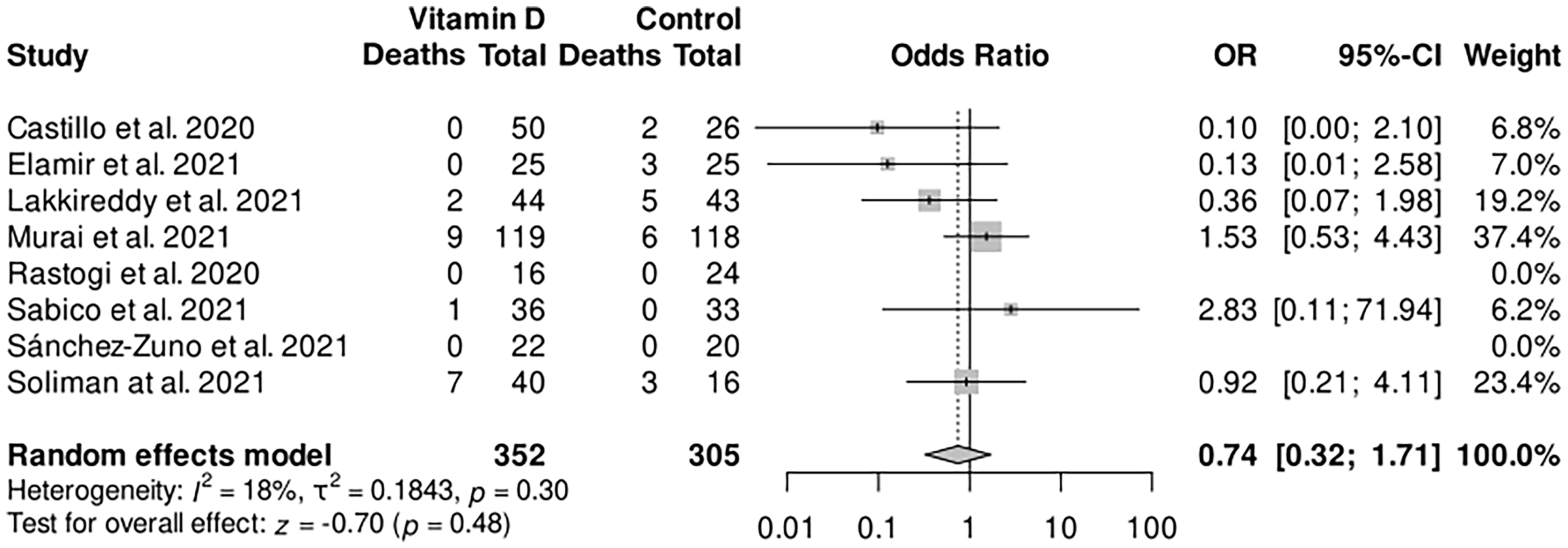
Forest plot random-effects model for the association of vitamin D supplementation and the main outcome mortality. CI, confidence interval; OR, odds ratio.

The primary analysis of mortality in the vitamin D group compared with the control group was assessed, revealing trends of reduced mortality in the intervention group, although it was not statistically significant [odds ratio (OR) 0.74, 95% confidence interval (CI) 0.32–1.71; **Figure 2**]. The mortality rate and the count of deaths were reported in all of the eight trials included: no deaths occurred in two trials, while the highest mortality rate was observed in the Soliman et al. trial being 17.86% among all patients; and none of the trials reported a statistically significant effect of vitamin D on mortality themselves.

**Figure 3:**
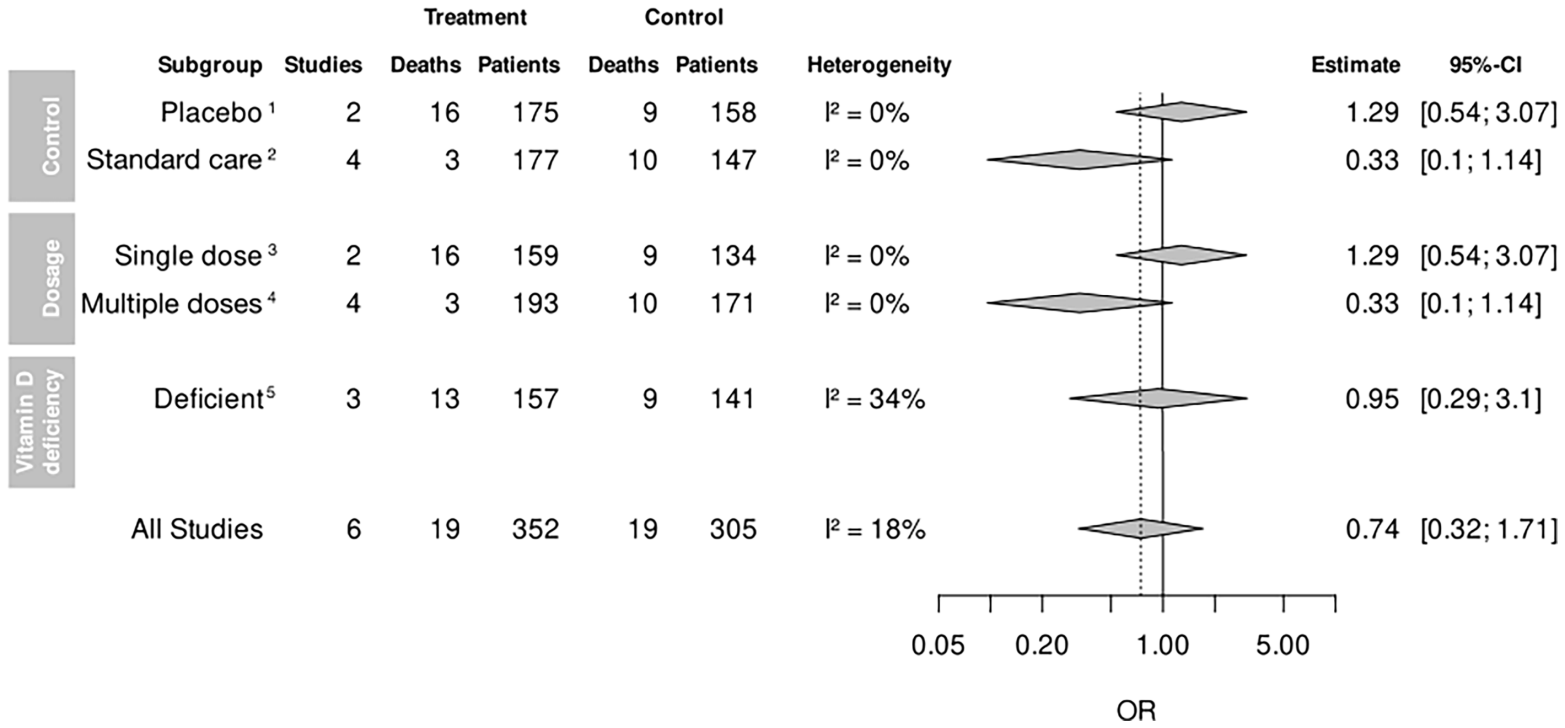
Mortality among subgroup analyses. CI, confidence interval; OR, odds ratio. ^1^Control group receiving a placebo. ^2^Control group receiving standard of care. ^3^Patients receiving a single dose of vitamin D once. ^4^Patients receiving more than one dose of vitamin D. ^5^Patients with confirmed vitamin D deficiency below 30 or 20 ng/ml at baseline.

Subgroup analysis comparing vitamin D supplementation to placebo did not show a beneficial effect on mortality, with an OR of 1.29 (95% CI 0.54–3.07; **Figure 3**), compared with standard of care in which lower odds were observed (OR 0.33, 95% CI 0.1–1.14), but no significance was reached in either of the subgroups. The subgroup receiving multiple dosages of vitamin D was associated with a trend of lower mortality (OR 0.33, 95% CI 0.1–1.14), by analyzing the same reported events as in the standard-of-care subgroup. Other studies administrating multiple doses of vitamin D did not report any deaths. For the patients receiving a single bolus of vitamin D compared with the control group, no beneficial effect was observed, with the same two studies included as for the placebo subgroup. Furthermore, the vitamin D deficiency subanalysis did not show an effect of vitamin D on mortality (OR 0.95, 95% CI 0.29–3.1) among all patients, with confirmed vitamin D serum levels below 30 or 20 ng/ml at baseline. Overall, subgroup analyses failed to reach statistically significant effects.

**Figure 4:**
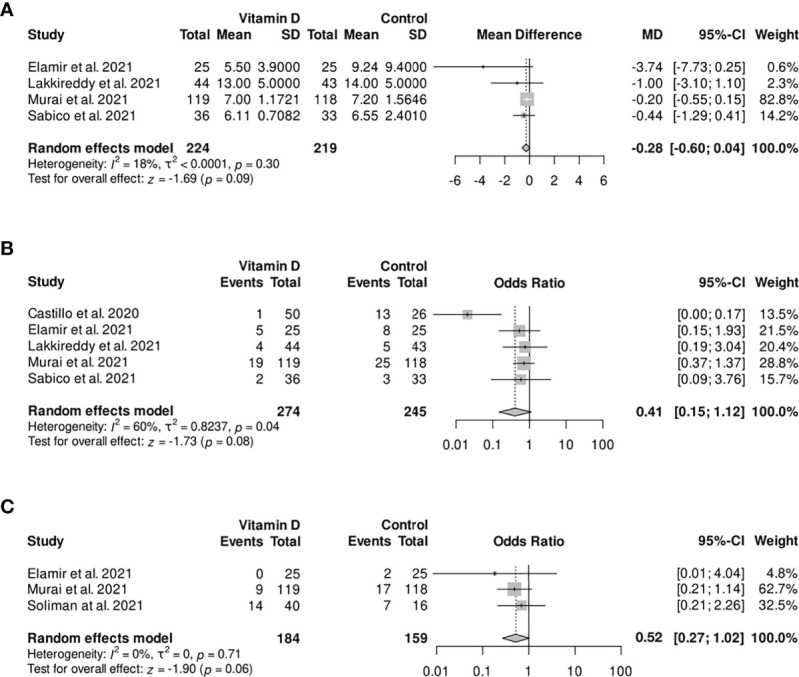
Forest plot random-effect model of various outcomes: **(A)** length of hospitalization; **(B)** need for ICU admission; **(C)** need for mechanical ventilation. CI, confidence interval; MD, mean difference; OR, odds ratio; SD, standard deviation.

By analyzing further clinical outcomes, no statistical significance was observed. However, the length of hospitalization tended to be shorter in the vitamin D group compared with the control group [mean difference (MD) −0.28, 95% CI −0.60 to 0.04; **Figure 4**]. It should be noted that the Murai et al. study was weighted 82.8% in this analysis. The random-effects model for the need of ICU admission is showing a less frequent admission to ICU in the intervention group (OR 0.41, 95% CI 0.15–1.12), while vitamin D supplementation was associated with lower odds for the need of mechanical ventilation compared with the control group (OR 0.52, 95% CI 0.27–1.02).

For the length of hospitalization and the need for ICU admission, further subgroups were formed, analyzing the trials administering repeated dosages of vitamin D supplementation only. In this process, no statistically significant effects of vitamin D supplementation compared with the control group were observed, with a trend of shorter hospitalization (MD −0.64, 95% CI −1.42 to 0.13) and less frequent admission to the ICU (OR 0.30, 95% CI 0.07–1.35; **Table 2**). None of these results were statistically significant.

**Table 2:** Outcomes in trials administrating vitamin D repeatedly.

Outcome	Studies	Events/treatment group	Events/control group	Heterogeneity	OR	95% CI
Mortality	6	3/193	10/171	0%	0.33	[0.11–1.14]
ICU admission	4	12/155	29/127	66%	0.30	[0.09–3.76]
Outcome	Studies	Treatment group	Control group	Heterogeneity	MD	95% CI
Length of hospitalization	3	105	101	24%	−0.64	[−1.42 to 0.41]

CI, confidence interval; MD, mean difference; OR, odds ratio.

Significant increases in vitamin D serum levels were assessed by analyzing the five trials reporting on vitamin D levels (**Supplementary Figure 3**), while three trials did not measure vitamin D serum levels. Thus, it is difficult to relate vitamin D concentrations with reported clinical outcomes.

### Publication bias and certainty of evidence

Publication bias was assessed using funnel plots with no significant risk of biases as revealed by Begg’s correlation test and Egger’s regression (**Supplementary Figure 10**). The overall quality of evidence was low to moderate, based on the GRADE profile that is available in **Supplementary Table 4**.

## Discussion

To our knowledge, this is the first study specifically focusing on dosing regimens of vitamin D in RCTs in the context of COVID-19 by performing further subgroup analyses. Previously published meta-analyses by Szarpak et al. and Rawat et al. reported similar results to our main outcomes, not reaching a statistical significance for the effect of vitamin D supplementation on abrogating COVID-19-related complications. However, they included quasi-experimental and non-randomized trials, as only three RCTs were available at the time of their final search (26, 27). In contrast, other meta-analyses reported significantly lower rates of adverse outcomes such as the one by Pal et al. (28). They revealed a significantly less frequent admission to the ICU and lower rates of mortality in patients receiving vitamin D supplementation by analyzing RCTs and observational studies. Vitamin D supplementation given before the diagnosis of COVID-19 did not show any benefit. The meta-analysis by Nikniaz et al. reported significantly lower rates of mortality in patients receiving vitamin D supplementation by analyzing two RCTs and one quasi-experimental study, and Shah et al. showed a significantly reduced need for ICU admission by analyzing two RCTs and one observational study (29, 30). Tentolouris et al. and Hosseini et al. also depicted significantly reduced needs for ICU admission, while Beran et al. showed significant effects of vitamin D for the length of hospitalization and the need for mechanical ventilation (29, 31, 32). Overall, these meta-analyses included other types of clinical trials on vitamin D supplementation, while we chose to focus on high-quality studies to limit the risk of bias. Varikasuvu et al. focused on RCTs and were able to show significant effects of vitamin D regarding PCR positivity and COVID-19 severity, but not for COVID-19-related mortality (33). They included fewer trials than we did with a total of six RCTs and only two for PCR positivity and assessed COVID-19 severity by analyzing the rate of ICU admission, need for mechanical ventilation, and severity of symptoms as one outcome.

Studies on other respiratory tract infections, conducted before the emergence of SARS-CoV-2, reported lower rates of acute respiratory infections in patients receiving vitamin D supplementation (34–36). One meta-analysis on vitamin D supplementation to prevent acute respiratory tract infections performed subgroup analyses for repeated doses of vitamin D, showing lower rates of respiratory tract infections when vitamin D supplementation was administered on a daily basis (1). In the meta-analysis by D’Ecclesiis, which included all kinds of studies, additional boluses and higher doses had no stronger effects (37).

Overall, previous studies generated heterogeneous results. While some report the protective effects of vitamin D supplementation on mortality and the need for ICU admission, others show no significant association between vitamin D supplementation and COVID-19-related outcomes. However, most studies have several limitations such as the inclusion of low-quality trials, trials offering additional supplementation other than vitamin D (34, 35), non-exclusive focus on COVID-19 (1, 36), missing information on vitamin D serum levels, heterogeneous baseline characteristics of included studies, and varying dosing and administration regimens of vitamin D supplementation. Overall, the quality of trials focusing on vitamin D and COVID-19 is an important issue that obviously affects the quality of published results. Importantly, the *Lancet* recently retracted a preprint article about a trial also investigating the effect of vitamin D treatments on COVID-19 and related outcomes as there was a series of mistakes made by the authors in the qualification of the study and its description (38).

Our analyses revealed no statistically significant findings for our main outcomes and subgroup analyses, but undoubtedly stronger effects were observed when vitamin D supplementation was administered repeatedly. However, it should be considered that the RCTs administrating multiple dosages of vitamin D without a placebo control are of lower quality than RCTs with placebo. The trials comparing vitamin D supplementation with placebo are only weighted in the single-bolus subgroup in this analysis. Therefore, placebo-controlled RCTs are required to draw more robust outcomes on the effect of multiple dosages of vitamin D supplementation in COVID-19 disease.

The main strength of our study is that we included only high-quality studies by limiting the eligible studies to RCTs comparing vitamin D to placebo or standard of care, while previous meta-analyses additionally or only analyzed observational studies. Secondly, we focus on trials investigating vitamin D supplementation in the context of COVID-19 as data from other respiratory infections may not be applicable, compared with meta-analyses assessing the effect of vitamin D supplementation on respiratory tract infections in general (1, 34–36). Thirdly, we augmented our search results by contacting trial investigators for unpublished data. Finally, we conducted an analysis on vitamin D supplementation administered repeatedly to limit the fluctuation of vitamin D serum levels, which is a novel aspect in this context, only investigated by previous meta-analyses performed before the emergence of SARS-CoV-2 (1). Hence, we were able to investigate the effect of a continuous increase of serum concentrations on clinical outcomes.

Our study also has some limitations. Firstly, only a small number of trials passing our inclusion criteria were available. In those trials, only a small number of outcome events were reported, leading to a risk of overestimation of the true intervention effect for assessed outcomes. Secondly, studies were quite heterogeneous using different dosing regimens of vitamin D supplementation. Moreover, the different study locations limit the generalizability of our findings to those settings, while the baseline characteristics of the participants included in individual RCTs were heterogeneous, with no adjustments for vitamin D serum levels. Thirdly, studies were not always evaluating the same primary outcome, or information on clinical outcomes was missing. Data especially on vitamin D serum levels and timing of follow-up measurements were not reported in all trials. Even after contacting trial investigators, not all information was available. Finally, some risk of bias was detected for the included studies (**Supplementary Figure 2**).

There is an urgent need for further investigation demonstrating the effect of vitamin D supplementation in the context of COVID-19, as sufficient vitamin D serum concentrations may help protect people from severe disease progression all over the world. The major challenge lies in assessing the relationship between vitamin D serum concentrations reached by supplementation and the occurrence of SARS-CoV-2 infection as well as disease-related clinical outcomes. Currently, there are more than 50 ongoing clinical trials on vitamin D supplementation in COVID-19 patients registered on ClinicalTrials.gov (39). However, when looking at their study protocols, 94% of them (**Supplementary Table 5**) may have difficulties to prove the beneficial effects of vitamin D serum concentrations reached by supplementation on COVID-19-related outcomes. Consistent monitoring of vitamin D serum levels is necessary, including a run-in period and the adjustment of baseline values between the intervention and the control group in the randomization process. The importance of adjusted vitamin D serum levels can be demonstrated by looking at the trial performed by Soliman et al. with the highest concentrations of vitamin D being measured in the control group at baseline (25), leading to a high risk of misclassification bias and reduced power. Furthermore, the study size must be chosen wisely to be able to show the significant effects of vitamin D supplementation, since participants in the control group can produce vitamin D endogenously as well. The period of investigation, including the duration of vitamin D supplementation and time until the follow-up assessments, needs to be of adequate length to reach sufficient vitamin D serum levels. Since our analysis showed a tendency for a harmful effect of a single bolus of vitamin D, vitamin D supplementation should be administered repeatedly (e.g., daily) rather than once. Considering these suggestions, only three of the ongoing trials, based on their study protocol, could be helpful to assess the effect of vitamin D supplementation for the treatment of COVID-19.

Interestingly, vitamin D supplementation can affect vitamin K levels by influencing vitamin D-dependent proteins and potentially can induce vitamin K deficiency (40, 41). As previous studies have demonstrated that a low vitamin K concentration is associated with higher risks of developing severe COVID-19 and increased IL-6 levels, undoubtedly, further research is needed to investigate the levels of vitamin K in the context of vitamin D insufficiency as well as the role of vitamin D supplementation in vitamin K levels and its related outcomes in the COVID-19 population (42–45).

Overall, a focus on reaching sufficient vitamin D serum levels is crucial, and not only in the context of COVID-19 vitamin D supplementation may be important to help improve immune function globally. With all age groups being affected, the risk of insufficient vitamin D status is prevalent worldwide. Multiple factors such as age, gender, ethnicity, or limited sun exposure influence vitamin D metabolism and can result in insufficient vitamin D serum concentrations. It is important to investigate vitamin D supplementation regimens as a safe and widely available supplement to reduce the prevalence of vitamin D deficiency globally and establish supplementation programs for people of high risk (6, 46, 47). Over the last decade, a successful nutrition policy in Finland resulted in improved vitamin D status in adult people nationally. Finland also reports low rates of SARS-CoV-2 infections and COVID-19-related deaths compared with the United States of America and Europe, even with the lowest rates of SARS-CoV-2 infections in Europe in November 2020. However, if vitamin D sufficiency is one of the multiple factors influencing the prevalence of COVID-19 in Finland and may help improve the global situation, further investigations are required (46, 48).

## Conclusion

In conclusion, our findings indicate that vitamin D supplementation is associated with the trend of reducing COVID-19-related mortality and clinical severity, especially in patients receiving repeated vitamin D doses, when vitamin D was given after the diagnosis of COVID-19. Repeated administration of vitamin D supplementation could be helpful to reach sufficient serum levels and improve immune function and, thus, COVID-19-related complications. The available evidence to date is of low quality with heterogeneous findings. Therefore, it is impossible to demonstrate the immunological benefit of vitamin D supplementation in this context yet. Hence, further investigations are required, including updated analyses of ongoing and future RCTs.

## Data availability statement

The raw data supporting the conclusions of this article will be made available by the authors, without undue reservation.

## Author contributions

LK, HK, CS, and PF conceptualized the study. LK and HK performed the literature search. LK, HK, BH, and RR performed the study screening and selection with the support of KP. LK and HK extracted and verified the study data. CT performed the analysis. LK, HK, CS, and PF wrote the first draft of the manuscript. All authors had access to all data in the study, contributed to the writing process, and approved the published version of the manuscript.

## Funding

The authors declare that this study received funding from the Universities Giessen and Marburg Lung Center (UGMLC), German Center for Lung Research (DZL), University Hospital Giessen and Marburg (UKGM) research funding according to article 2, section 3 cooperation agreement, Deutsche Forschungsgemeinschaft (DFG, German Research Foundation) - SFB 1021 (Project-ID 197785619), KFO 309 (P10), and SK 317/1-1 (Project ID 428518790) and from the Foundation for Pathobiochemistry and Molecular Diagnostics. The funders were not involved in the study design, collection, analysis, interpretation of data, the writing of this article, or the decision to submit it for publication.

## Conflict of interest

CS received consultancy fees and research funding from Hycor Biomedical, Bencard Allergie, and Thermo Fisher Scientific and research funding from Mead Johnson Nutrition MJN.

The remaining authors declare that the research was conducted in the absence of any commercial or financial relationships that could be construed as a potential conflict of interest.

## Publisher’s note

All claims expressed in this article are solely those of the authors and do not necessarily represent those of their affiliated organizations, or those of the publisher, the editors and the reviewers. Any product that may be evaluated in this article, or claim that may be made by its manufacturer, is not guaranteed or endorsed by the publisher.

## References

[1] Jolliffe DA Camargo CA Jr Sluyter JD Aglipay M Aloia JF Ganmaa D. Vitamin D supplementation to prevent acute respiratory infections: A systematic review and meta-analysis of aggregate data from randomised controlled trials. Lancet Diabetes Endocrinol (2021) 9(5):276–92. doi: 10.1016/s2213-8587(21)00051-6 33798465

[2] Prietl B Treiber G Pieber TR Amrein K. Vitamin d and immune function. Nutrients (2013) 5(7):2502–21. doi: 10.3390/nu5072502 PMC3738984 23857223

[3] Greiller CL Martineau AR. Modulation of the immune response to respiratory viruses by vitamin d. Nutrients (2015) 7(6):4240–70. doi: 10.3390/nu7064240 PMC4488782 26035247

[4] Bui L Zhu Z Hawkins S Cortez-Resendiz A Bellon A. Vitamin d regulation of the immune system and its implications for COVID-19: A mini review. SAGE Open Med (2021) 9:20503121211014073. doi: 10.1177/20503121211014073 34046177 PMC8135207

[5] Aranow C. Vitamin d and the immune system. J Investig Med (2011) 59(6):881–6. doi: 10.2310/JIM.0b013e31821b8755 PMC3166406 21527855

[6] Amrein K Scherkl M Hoffmann M Neuwersch-Sommeregger S Köstenberger M Tmava Berisha A. Vitamin d deficiency 2.0: An update on the current status worldwide. Eur J Clin Nutr (2020) 74(11):1498–513. doi: 10.1038/s41430-020-0558-y PMC7091696 31959942

[7] Petrelli F Luciani A Perego G Dognini G Colombelli PL Ghidini A. Therapeutic and prognostic role of vitamin d for COVID-19 infection: A systematic review and meta-analysis of 43 observational studies. J Steroid Biochem Mol Biol (2021) 211:105883. doi: 10.1016/j.jsbmb.2021.105883 33775818 PMC7997262

[8] Page MJ McKenzie JE Bossuyt PM Boutron I Hoffmann TC Mulrow CD. The PRISMA 2020 statement: An updated guideline for reporting systematic reviews. BMJ (2021) 372:n71. doi: 10.1136/bmj.n71 33782057 PMC8005924

[9] Higgins JPT Thomas J Chandler J Cumpston M Li T Page MJ. Cochrane Handbook for Systematic Reviews of Interventions. 2nd Edition (Chichester (UK): John Wiley & Sons) (2019).

[10] WHO. COVID-19: Case definitions. World Health Organization: WHO (2020).

[11] Sterne JAC Savović J Page MJ Elbers RG Blencowe NS Boutron I. RoB 2: A revised tool for assessing risk of bias in randomised trials. Bmj. (2019) 366:14898. doi: 10.1136/bmj.l4898 31462531

[12] Balduzzi S Rücker G Schwarzer G. How to perform a meta-analysis with r: a practical tutorial. Evid Based Ment Health (2019) 22(4):153–60. doi: 10.1136/ebmental-2019-300117 PMC10231495 31563865

[13] Team RC. R: A language and environment for statistical computing. (Vienna, Austria: R Foundation for Statistical Computing) (2021).

[14] Mantel N Haenszel W. Statistical aspects of the analysis of data from retrospective studies of disease. J Natl Cancer Inst (1959) 22(4):719–48. doi: 10.1093/jnci/22.4.719 13655060

[15] Egger M Davey Smith G Schneider M Minder C. Bias in meta-analysis detected by a simple, graphical test. Bmj (1997) 315(7109):629–34. doi: 10.1136/bmj.315.7109.629 PMC2127453 9310563

[16] Begg CB Mazumdar M. Operating characteristics of a rank correlation test for publication bias. Biometrics (1994) 50(4):1088–101. doi: 10.2307/2533446 7786990

[17] GRADEpro GDT. GRADEpro guideline development tool. McMaster University and Evidence Prime (2021).

[18] Castillo ME Costa LME Barrios JMV Díaz JFA Miranda JL Bouillon R. Effect of calcifediol treatment and best available therapy versus best available therapy on intensive care unit admission and mortality among patients hospitalized for COVID-19: A pilot randomized clinical study. J Steroid Biochem Mol Biol (2020) 203:105751. doi: 10.1016/j.jsbmb.2020.105751 32871238 PMC7456194

[19] Elamir YM Amir H Lim S Rana YP Lopez CG Feliciano NV. A randomized pilot study using calcitriol in hospitalized COVID-19 patients. Bone (2022) 154:116175. doi: 10.1016/j.bone.2021.116175 34508882 PMC8425676

[20] Lakkireddy M Gadiga SG Malathi RD Karra ML Raju I Ragini. Impact of daily high dose oral vitamin d therapy on the inflammatory markers in patients with COVID 19 disease. Sci Rep (2021) 11(1):10641. doi: 10.1038/s41598-021-90189-4 34017029 PMC8138022

[21] Murai IH Fernandes AL Sales LP Pinto AJ Goessler KF Duran CSC. Effect of a single high dose of vitamin D3 on hospital length of stay in patients with moderate to severe COVID-19: A randomized clinical trial. Jama (2021) 325(11):1053–60. doi: 10.1001/jama.2020.26848 PMC7890452 33595634

[22] Rastogi A Bhansali A Khare N Suri V Yaddanapudi N Sachdeva N. Short term, high-dose vitamin d supplementation for COVID-19 disease: A randomised, placebo-controlled, study (SHADE study). Postgrad Med J (2020) 98(1156):87–90. doi: 10.1136/postgradmedj-2020-139065 33184146

[23] Sabico S Enani MA Sheshah E Aljohani NJ Aldisi DA Alotaibi NH. Effects of a 2-week 5000 IU versus 1000 IU vitamin D3 supplementation on recovery of symptoms in patients with mild to moderate covid-19: A randomized clinical trial. Nutrients (2021) 13(7):2170. doi: 10.3390/nu13072170 34202578 PMC8308273

[24] Sánchez-Zuno GA González-Estevez G Matuz-Flores MG Macedo-Ojeda G Hernández-Bello J Mora-Mora JC. Vitamin d levels in COVID-19 outpatients from Western Mexico: Clinical correlation and effect of its supplementation. J Clin Med (2021) 10(11):2378. doi: 10.3390/jcm10112378 PMC8198869 34071293

[25] Soliman AR Abdelaziz TS Fathy A. Impact of vitamin d therapy on the progress COVID-19: Six weeks follow-up study of vitamin d deficient elderly diabetes patients. Proc Singapore Healthcare (2021) 0(0):20101058211041405. doi: 10.1177/20101058211041405

[26] Szarpak L Filipiak KJ Gasecka A Gawel W Koziel D Jaguszewski MJ. Vitamin d supplementation to treat SARS-CoV-2 positive patients. evidence from meta-analysis. Cardiol J (2021) 29(2):188–96. doi: 10.5603/CJ.a2021.0122 PMC9007480 34642923

[27] Rawat D Roy A Maitra S Shankar V Khanna P Baidya DK. "Vitamin d supplementation and COVID-19 treatment: A systematic review and meta-analysis". Diabetes Metab Syndr (2021) 15(4):102189. doi: 10.1016/j.dsx.2021.102189 34217144 PMC8236412

[28] Pal R Banerjee M Bhadada SK Shetty AJ Singh B Vyas A. Vitamin d supplementation and clinical outcomes in COVID-19: A systematic review and meta-analysis. J Endocrinol Invest (2022) 45(1):53–68. doi: 10.1007/s40618-021-01614-4 34165766 PMC8223190

[29] Nikniaz L Akbarzadeh MA Hosseinifard H Hosseini M-S. The impact of vitamin d supplementation on mortality rate and clinical outcomes of COVID-19 patients: A systematic review and meta-analysis. Pharm Sci (2021) 27:S1–12. doi: 10.34172/ps.2021.13

[30] Shah K Saxena D Mavalankar D. Vitamin d supplementation, COVID-19 and disease severity: A meta-analysis. Qjm (2021) 114(3):175–81. doi: 10.1093/qjmed/hcab009 PMC7928587 33486522

[31] Tentolouris N Samakidou G Eleftheriadou I Tentolouris A Jude EB. The effect of vitamin d supplementation on mortality and intensive care unit admission of COVID-19 patients. A systematic review, meta-analysis and meta-regression. Diabetes Metab Res Rev (2022) 38(4):e3517. doi: 10.1002/dmrr.3517 34965318 PMC9015406

[32] Beran A Mhanna M Srour O Ayesh H Stewart JM Hjouj M. Clinical significance of micronutrient supplements in patients with coronavirus disease 2019: A comprehensive systematic review and meta-analysis. Clin Nutr ESPEN (2022) 48:167–77. doi: 10.1016/j.clnesp.2021.12.033 PMC8755558 35331487

[33] Varikasuvu SR Thangappazham B Vykunta A Duggina P Manne M Raj H. COVID-19 and vitamin d (Co-VIVID study): A systematic review and meta-analysis of randomized controlled trials. Expert Rev Anti Infect Ther (2022) 20(6):907–13. doi: 10.1080/14787210.2022.2035217 PMC8862170 35086394

[34] Abioye AI Bromage S Fawzi W. Effect of micronutrient supplements on influenza and other respiratory tract infections among adults: A systematic review and meta-analysis. BMJ Glob Health (2021) 6(1):e003176. doi: 10.1136/bmjgh-2020-003176 PMC7818810 33472840

[35] Vlieg-Boerstra B de Jong N Meyer R Agostoni C De Cosmi V Grimshaw K. Nutrient supplementation for prevention of viral respiratory tract infections in healthy subjects: A systematic review and meta-analysis. Allergy (2021) 77(5):1373–88. doi: 10.1111/all.15136 34626488

[36] Martineau AR Jolliffe DA Hooper RL Greenberg L Aloia JF Bergman P. Vitamin d supplementation to prevent acute respiratory tract infections: Systematic review and meta-analysis of individual participant data. Bmj (2017) 356:i6583. doi: 10.1136/bmj.i6583 28202713 PMC5310969

[37] D'Ecclesiis O Gavioli C Martinoli C Raimondi S Chiocca S Miccolo C. Vitamin d and SARS-CoV2 infection, severity and mortality: A systematic review and meta-analysis. PloS One (2022) 17(7):e0268396. doi: 10.1371/journal.pone.0268396 35793346 PMC9258852

[38] Nogués X Ovejero D Quesada-Gomez JM Bouillon R Arenas D Pascual J. Calcifediol treatment and COVID-19-Related outcomes. Preprints Lancet (2021) 106(10):4017–27 doi: 10.1210/clinem/dgab405 PMC8344647 34097036

[39] NIH. U.S. National Library of Medicine. Available at: https://clinicaltrials.gov. 10.1080/15360280801989377 28792816

[40] Fraser JD Price PA. Induction of matrix gla protein synthesis during prolonged 1,25-dihydroxyvitamin D3 treatment of osteosarcoma cells. Calcif Tissue Int (1990) 46(4):270–9. doi: 10.1007/bf02555007 2108798

[41] van Ballegooijen AJ Beulens JWJ Kieneker LM de Borst MH Gansevoort RT Kema IP. Combined low vitamin d and K status amplifies mortality risk: a prospective study. Eur J Nutr (2021) 60(3):1645–54. doi: 10.1007/s00394-020-02352-8 PMC7987611 32808059

[42] Linneberg A Kampmann FB Israelsen SB Andersen LR Jørgensen HL Sandholt H. The association of low vitamin K status with mortality in a cohort of 138 hospitalized patients with COVID-19. Nutrients (2021) 13(6):1985. doi: 10.3390/nu13061985 PMC8229962 34207745

[43] Desai AP Dirajlal-Fargo S Durieux JC Tribout H Labbato D McComsey GA. Vitamin K & d deficiencies are independently associated with COVID-19 disease severity. Open Forum Infect Dis (2021) 8(10):ofab408. doi: 10.1093/ofid/ofab408 34642636 PMC8344499

[44] Dofferhoff ASM Piscaer I Schurgers LJ Visser MPJ van den Ouweland JMW de Jong PA. Reduced vitamin K status as a potentially modifiable risk factor of severe coronavirus disease 2019. Clin Infect Dis (2021) 73(11):e4039–e46. doi: 10.1093/cid/ciaa1258 PMC7499546 32852539

[45] Visser MPJ Dofferhoff ASM van den Ouweland JMW van Daal H Kramers C Schurgers LJ. Effects of vitamin d and K on interleukin-6 in COVID-19. Front Nutr (2021) 8:761191. doi: 10.3389/fnut.2021.761191 35111793 PMC8801698

[46] Raulio S Erlund I Männistö S Sarlio-Lähteenkorva S Sundvall J Tapanainen H. Successful nutrition policy: improvement of vitamin d intake and status in Finnish adults over the last decade. Eur J Public Health (2017) 27(2):268–73. doi: 10.1093/eurpub/ckw154 28339536

[47] Roth DE Abrams SA Aloia J Bergeron G Bourassa MW Brown KH. Global prevalence and disease burden of vitamin d deficiency: A roadmap for action in low- and middle-income countries. Ann N Y Acad Sci (2018) 1430(1):44–79. doi: 10.1111/nyas.13968 30225965 PMC7309365

[48] Höppner S. Why is Finland coping so well with the coronavirus crisis? DW: Deutsche Welle (2020).

