# Supplementary material for: Vitamin D supplementation for the treatment of COVID-19: A systematic review and meta-analysis of randomized controlled trials
Source: Front Immunol. 2022 Oct 31;13:1023903. doi: 10.3389/fimmu.2022.1023903 (PMC9659578; doi:10.3389/fimmu.2022.1023903)
Supplement: Supplementary file 1 [file DataSheet_1.docx]

**Vitamin D supplementation for treatment of COVID-19: a systematic review and meta-analysis of randomized controlled trials**

Lara S. Kümmel^†,1^, Hanna Krumbein^†,1^, Paraskevi C. Fragkou^2^, Ben L. Hünerbein^1^, Rieke Reiter^1^, Konstantinos A. Papathanasiou^3^, Clemens Thölken^4^, Scott T. Weiss^5^, Harald Renz^1^, Chrysanthi Skevaki^1,*^

^†^These authors share first authorship

^1^ Institute of Laboratory Medicine, Universities of Giessen and Marburg Lung Center (UKGMLC), Philipps Universität Marburg, German Center for Lung Research (DZL) Marburg, Marburg, Germany

^2^ First Department of Critical Care Medicine and Pulmonary Services, Evangelismos Hospital, Medical School of Athens, National and Kapodistrian University of Athens, Athens, Greece

^3^ Medical School, National and Kapodistrian University of Athens, Athens, Greece

^4^ Institute of Medical Bioinformatics and Biostatistics, Medical Faculty, Philipps University of Marburg, Marburg, Germany

^5^ Channing Division of Network Medicine, Department of Medicine, Brigham and Women’s Hospital, Harvard Medical School, Boston, MA, USA

^*^**Correspondence**

[Chrysanthi.Skevaki@uk-gm.de](mailto:Chrysanthi.Skevaki@uk-gm.de)

Supplementary Tables

**Supplementary Table 1** Search strings

**Supplementary Table 2** PICO algorithm

**Supplementary Table 3** predefined definitions

**Supplementary Table 4** GRADE evidence profile

**Supplementary Table 5.1** Ongoing trials registered on ClinicalTrials.gov

**Supplementary Table 5.2** Analysis of ongoing trials registered on ClinicalTrials.gov

**Supplementary Table 6** PRISMA Checklist

Supplementary Figures

**Supplementary Figure 1** Proportion of gender per study, with an over proportion of 43.15% being female

**Supplementary Figure 2** Overall results of the risk of bias assessment

**Supplementary Figure 3** Forest plot showing the increase of vitamin D serum levels in patients, after receiving the intervention

**Supplementary Figure 4** Forest plot with subgroup analysis showing the effect of vitamin D supplementation compared to placebo and standard of care

**Supplementary Figure 5** Forest plot with subgroup analysis showing the effect of single or repeated dosages of vitamin D on mortality

**Supplementary Figure 6** Forest plot with subgroup analysis showing the effect of vitamin D supplementation on mortality in patients with confirmed vitamin D deficiency (as defined by study investigators)

**Supplementary Figure 7** Forest plot with subgroup analysis showing the effect of repeated vitamin D supplementation on the length of hospitalization

**Supplementary Figure 8** Forest plot with subgroup analysis showing the effect of repeated vitamin D supplementation on the need for ICU admission

**Supplementary Figure 9** Forest Correlation of vitamin D serum levels and various outcomes weighted by study size: **A** Mortality; **B** Length of hospitalization; **C** ICU admission

**Supplementary Figure 10** Funnel plot for mortality assessing risk of publication bias

References

**Supplementary Table 1** Search strings

| **Concept** | **Search strings** |
| --- | --- |
| All terms within each concept were combined with OR, all concepts with AND. | |
| 1. COVID-19 | ((((((((((((((covid) OR (covid*)) OR (covid-19)) OR (covid 19)) OR (coronavirus)) OR (coronavirus disease)) OR (2019 ncov)) OR (ncov)) OR (sars-cov-2)) OR (sars cov 2)) OR (sars coronavirus 2)) OR (sars sov 2 infection)) OR (severe acute respiratory syndrome)) OR (acute respiratory syndrome coronavirus)) OR (covid 19 virus disease*) |
| 1. Vitamin D supplementation | (((((((((((((vitamin d) OR (vitamin d3)) OR (vit d)) OR (vit d3)) OR (calciferol)) OR (cholecalciferol)) OR (calcidiol)) OR (calcitriol)) OR (25 hydroxyvitamin d)) OR (25 hydroxyvitamin d3)) OR (25 hydroxycalciferol)) OR (1,25 dihydroxyvitamin d)) OR (1,25 dihydroxyvitamin d3)) OR (calcifediol) |
| 1. Language | (German) OR (English) |
| 1. Publication date | 01/11/2021 – 17/09/2021 |

**Supplementary Table 2** Pico algorithm

|  | **Inclusion criteria** | **Exclusion criteria** |
| --- | --- | --- |
| Participants | - Adults and children with no restriction on age, gender or ethnicity - Inpatients and outpatients - With known SARS-COV-2 status (diagnosis defined by WHO definitions) | - Criteria for definition of SARS-CoV-2 diagnosis not fulfilled |
| Intervention | - Vitamin D3 supplementation - With no restriction on the dosage | - Missing information on vitamin D supplementation |
| Comparison | - Placebo - Standard of care - No treatment | - No comparator - Control group receiving same dose of vitamin D |
| Outcomes | - Mortality and various clinical outcomes (e.g., length of hospitalization, ICU admission, mechanical ventilation) - Vitamin D serum levels | - No results available |
| Studies | - Randomized controlled trials - Published in English or German language | - Any other kind of study - Published in other language than English or German |

**Supplementary Table 3** Predefined definitions

| **COVID-19 definition**  By WHO COVID-19: Case definition | *Confirmed case of SARS-CoV-2 infection:* (i) person with positive Nucleic Acid Amplification Test; (ii) positive SARS-CoV-2 Antigen-RDT AND meeting either the probable case definition or suspect criteria A OR B; (iii) asymptomatic person with a positive SARS-CoV-2 Antigen RDT who is a contact to a probable or confirmed case  *Probable case of SARS-CoV-2 infection:* (i) patient meeting clinical criteria below AND is a contact of a probable or confirmed case, or linked to a COVID-19 cluster; (ii) suspect case with chest imaging showing findings suggestive of COVID-19; (iii) person with recent onset of anosmia or ageusia; (iv) death, not otherwise explained, in an adult with respiratory distress preceding death AND contact to a probable or confirmed case, or linked to a COVID-19 cluster  *Suspected case of SARS-CoV-2 infection:* (i) person who meets clinical AND epidemiological criteria; (ii) patients with severe acute respiratory illness, (iii) asymptomatic person not meeting epidemiological criteria with a positive SARS-CoV-2 Antigen-RDT  Clinical Criteria: acute onset of fever AND cough; OR acute onset of any three or more of the following signs or symptoms (fever, cough, general weakness/fatigue, headache, myalgia, sore throat, coryza, dyspnoea, anoreaxia/nausea/vomiting, diarrhoea, altered mental status  Epidemiological Criteria: residing or working in area with high risk of transformation of the virus; residing or traveling to an area with community transmission; working in any helath care setting |
| --- | --- |
| **Disease severity**  By NIH: COVID-19 Treatment Guidelines | *„Asymptomatic or Presymptomatic Infection:* Individuals who test positive for SARS-CoV-2 using a virologic test (i.e., a nucleic acid amplification test [NAAT] or an antigen test) but who have no symptoms that are consistent with COVID-19.  *Mild Illness:* Individuals who have any of the various signs and symptoms of COVID-19 (e.g., fever, cough, sore throat, malaise, headache, muscle pain, nausea, vomiting, diarrhea, loss of taste and smell) but who do not have shortness of breath, dyspnea, or abnormal chest imaging.  *Moderate Illness:* Individuals who show evidence of lower respiratory disease during clinical assessment or imaging and who have an oxygen saturation (SpO2) ≥94% on room air at sea level.  *Severe Illness:* Individuals who have SpO2 <94% on room air at sea level, a ratio of arterial partial pressure of oxygen to fraction of inspired oxygen (PaO2/FiO2) <300 mm Hg, respiratory frequency >30 breaths/min, or lung infiltrates >50%.  *Critical Illness:* Individuals who have respiratory failure, septic shock, and/or multiple organ dysfunction.“ |
| **Vitamin D status**  By NIH: Vitamin D – Fact Sheet for Health Professionals | *Serum vitamin D concentrations:*  < 20 ng/mL inadequate for bone and overall health  > 20 ng/mL adequate for bone and overall health  > 50 ng/mL potential for adverse events |

(1-3)

**Supplementary Table 4** GRADE evidence profile

| **Certainty assessment** | | | | | | | **№ of patients** | | **Effect** | | **Certainty** | **Importance** |
| --- | --- | --- | --- | --- | --- | --- | --- | --- | --- | --- | --- | --- |
| **№ of studies** | **Study design** | **Risk of bias** | **Inconsistency** | **Indirectness** | **Imprecision** | **Other considerations** | **Vitamin D supplementation** | **Control** | **Relative (95% CI)** | **Absolute (95% CI)** |  |  |
| **Mortality (assessed with: Number of deaths)** | | | | | | | | | | | | |
| 8 | randomized trials | not serious | not serious | not serious | serious^a^ | none | 19/352 (5.4%) | 19/305 (6.2%) | **OR 0.74** (0.32 to 1.71) | **15 fewer per 1.000** (from 41 fewer to 40 more) | ⨁⨁⨁◯ Moderate |  |
| **Length of hospitalization (assessed with: Number of days hospitalized)** | | | | | | | | | | | | |
| 4 | randomized trials | not serious | not serious | not serious | serious^a^ | none | 224 | 219 | - | MD **0.28 days fewer** (0.6 fewer to 0.04 more) | ⨁⨁⨁◯ Moderate |  |
| **Need for ICU admission (assessed with: Number of ICU admissions)** | | | | | | | | | | | | |
| 5 | randomized trials | not serious | serious^b^ | not serious | serious^a^ | none | 31/274 (11.3%) | 54/245 (22.0%) | **OR 0.41** (0.15 to 1.12) | **117 fewer per 1.000** (from 180 fewer to 20 more) | ⨁⨁◯◯ Low |  |
| **Need for mechanical ventilation (assessed with: Number of mechanical ventilation needed)** | | | | | | | | | | | | |
| 3 | randomized trials | not serious | not serious | not serious | serious^a^ | none | 23/184 (12.5%) | 26/159 (16.4%) | **OR 0.52** (0.27 to 1.02) | **71 fewer per 1.000** (from 113 fewer to 3 more) | ⨁⨁⨁◯ Moderate |  |
| **Increase in vitamin D serum levels (assessed with: Baseline and follow-up serum levels)** | | | | | | | | | | | | |
| 5 | randomized trials | not serious | very serious^c^ | not serious | serious | dose response gradient | 255 | 255 | - | MD **30.43 ng/ml higher** (6.04 higher to 54.82 higher) | ⨁⨁◯◯ Low |  |
| **Subgroup analysis of mortality for single dosage of vitamin D supplementation (assessed with: Number of deaths)** | | | | | | | | | | | | |
| 2 | randomized trials | not serious | not serious | not serious | serious^a^ | none | 16/159 (10.1%) | 9/134 (6.7%) | **OR 1.29** (0.54 to 3.07) | **18 more per 1.000** (from 30 fewer to 114 more) | ⨁⨁⨁◯ Moderate |  |
| **Subgroup analysis of mortality for repeated dosage of vitamin D supplementation (assessed with: Number of deaths)** | | | | | | | | | | | | |
| 6 | randomized trials | not serious | not serious | not serious | serious^a^ | none | 3/193 (1.6%) | 10/171 (5.8%) | **OR 0.33** (0.10 to 1.14) | **38 fewer per 1.000** (from 52 fewer to 8 more) | ⨁⨁⨁◯ Moderate |  |
| **Subgroup analysis of mortality for patients with confirmed vitamin D deficiency (assessed with: Number of deaths)** | | | | | | | | | | | | |
| 4 | randomized trials | not serious | serious^d^ | not serious | serious | none | 13/157 (8.3%) | 9/141 (6.4%) | **OR 0.95** (0.29 to 3.10) | **3 fewer per 1.000** (from 44 fewer to 111 more) | ⨁⨁◯◯ Low |  |
| **Subgroup analysis of mortality for patients with vitamin D sufficiency (assessed with: Number of deaths)** | | | | | | | | | | | | |
| 5 | randomized trials | not serious | serious^e^ | not serious | serious | none | 10/252 (4.0%) | 11/222 (5.0%) | **OR 0.65** (0.14 to 3.07) | **17 fewer per 1.000** (from 42 fewer to 88 more) | ⨁⨁◯◯ Low |  |
| **Subgroup analysis of mortality in placebo controlled trials (assessed with: Number of deaths)** | | | | | | | | | | | | |
| 3 | randomized trials | not serious | not serious | not serious | serious^a^ | none | 16/175 (9.1%) | 9/158 (5.7%) | **OR 1.29** (0.54 to 3.07) | **15 more per 1.000** (from 25 fewer to 99 more) | ⨁⨁⨁◯ Moderate |  |
| **Subgroup analysis of mortality in trials compared to standard of care treatment (assessed with: Number of deaths)** | | | | | | | | | | | | |
| 5 | randomized trials | not serious | not serious | not serious | serious^a^ | none | 3/177 (1.7%) | 10/147 (6.8%) | **OR 0.33** (0.10 to 1.14) | **45 fewer per 1.000** (from 61 fewer to 9 more) | ⨁⨁⨁◯ Moderate |  |

Abbreviations: CI: confidence interval; MD: mean difference; OR: odds ratio

a. Low number of events

b. Heterogeneity of 60%

c. Heterogeneity of 100%

d. Heterogeneity of 34%

e. Heterogeneity of 40%

**Supplementary Table 5.1** Ongoing trials registered on ClinicalTrials.gov. The search was conducted on February 9, 2022 using the following search terms: “COVID-19 OR SARS-CoV-2” AND “vitamin D.” Duplicates, trials not investigating COVID-19 and trials not administrating vitamin D were excluded.

| **NCT** | **Status 09.02.2022** | **Trial Design** | **Country** | **Disease** | **Intervention** | **Control** | **Dose Vitamin D** | **Administration** | **Duration** | **Baseline Serum Level** | **Follow-up Serum Level** | **Sample Size Total** | **Assessment** |
| --- | --- | --- | --- | --- | --- | --- | --- | --- | --- | --- | --- | --- | --- |
| **Suggested:** |  | **RCT** |  | **COVID-19** | **Vitamin D only** | **Placebo or Standard Care** |  | **daily** | **≥ 1 month** | **Yes** | **Yes** | **> 50** |  |
| NCT04981743 | Recruting | RCT | Egypt | COVID-19 | Vitamin D | Standard Care (and others) | 2000 IU | daily |  |  |  | 100 |  |
| NCT04400890 | Terminated | RCT | USA | COVID-19 | Vitamin D (and Resveratrol) | Placebo | 100000 IU | single |  |  |  | 200 |  |
| NCT05092698 | Recruting | RCT | Russia | COVID-19 | Vitamin D | Placebo | 60000 IU | weekly | Until discharge | Yes |  | 100 |  |
| NCT04536298 | Recruting | RCT | USA | COVID-19 | Vitamin D | Placebo | 9600 followed by 3200 IU | daily | 28 days |  |  | 2700 |  |
| NCT04883203 | Completed | RCT | Tunisia | COVID-19 | Vitamin D | Placebo | 200000 IU | single |  |  |  | 130 |  |
| NCT04868903 | Recruting | RCT | USA | COVID-19 | Vitamin D | Differente doses | 400 IU, 4000 IU, or 10000 IU | daily | 9 months | Yes | Yes, four times | 2000 | Criteria fulfilled |
| NCT04552951 | Recruting | RCT | Spain | COVID-19 | Vitamin D | No treatment | 100000 IU | single |  |  |  | 80 |  |
| NCT05037253 | Completed | RCT | Russia | COVID-19 | Vitamin D | 2.000 IU daily | 50000 IU twice followed by 5000 IU daily | 5000 IU daily | 3 months | Yes | Yes | 128 | Criteria fulfilled |
| NCT05166005 | Active | RCT | Russia | COVID-19 | Vitamin D | 2.000 IU daily | 50000 IU twice | weekly |  | Yes | Yes | 350 |  |
| NCT04411446 | Completed | RCT | Argentina | COVID-19 | Vitamin D | Placebo | 100000 IU | once |  |  |  | 218 |  |
| NCT04482673 | Recruting | RCT | USA | COVID-19 | Vitamin D | Placebo | SARS-CoV-2 negative 6000 IU, positive plus three boluses 20000 IU | daily | 12 months |  |  | 140 |  |
| NCT05008003 | Not yet recruting | RCT | Pakistan | COVID-19 | Vitamin D | Standard Care | 800 IU | daily | 14 days |  |  | 100 |  |
| NCT04483635 | Terminated | RCT | Canada | COVID-19 | Vitamin D | Placebo | 100000 IU bolus, plus 10000 IU weekly | bolus plus weekly | 16 weeks |  |  | 34 |  |
| NCT04733625 | Completed | RCT | Egypt | COVID-19 with DM und vitamin D deficiency | Vitamin D | Placebo | 200000 IU | single |  |  |  | 56 |  |
| NCT04449718 | Completed | RCT | Brazil | COVID-19 | Vitamin D | Placebo | 200000 IU | single |  | Yes | Yes, twice | 240 |  |
| NCT04780061 | Recruting | RCT | Canada | COVID-19 | Vitamin D (and other supplements) | Placebo | 50000 IU |  |  |  |  | 200 |  |
| NCT05126602 | Completed | RCT | Indonesia | COVID-19 | Vitamin D | 1.000 IU | 10000 IU | daily | 2 weeks |  | Yes | 60 |  |
| NCT04952857 | Completed | RCT | India | COVID-19 with vitamin D deficiency | Vitamin D | Placebo | 6000 IU |  |  | Yes | Yes, three times | 90 |  |
| NCT04351490 | Withdrawn | RCT | France | COVID-19 | Vitamin D (and zinc) | Standard Care | 2000 IU | daily | 2 months |  |  | 0 |  |
| NCT04825093 | Not yet recruting | RCT | Spain | COVID-19, in pregnant women | Vitamin D | 400 IU | 2000 IU | daily | until delivery |  | Yes, continiousl | 500 |  |
| NCT04385940 | Recruting | RCT | Canada | COVID-19 | Vitamin D | 1.000 IU | 50000 IU |  |  | Yes |  | 64 |  |
| NCT04334005 | Not yet recruting | RCT | Spain | COVID-19 | Vitamin D | Standard Care | 25000 IU |  |  |  |  | 200 |  |
| NCT04636086 | Recruting | RCT | Belgium | COVID-19 | Vitamin D | Placebo | 25000 IU | daily, followed by weekly | 36 days | Yes | Yes, continiously | 100 |  |
| NCT04793243 | Completed | RCT | Mexico | COVID-19 | Vitamin D | No treatment | 10000 IU | daily | 14 days | Yes | Yes | 42 |  |
| NCT04502667 | Recruting | RCT | Mexico | COVID-19, in children | Vitamin D | No treatment | 1000 IU or 2000 IU when older | daily | until discharge | Yes | Yes | 40 |  |
| NCT04344041 | Completed | RCT | France | COVID-19 | Vitamin D | 50.000 IU | 400000 IU | single |  | Yes | Yes | 260 |  |
| NCT04407286 | Completed | Clinical Trial | USA | COVID-19 with vitamin D deficiency | Vitamin D | None | 10000 IU or 15000 IU when older | daily | 2 weeks, with low levels longer | Yes | Yes | 41 |  |
| NCT04459247 | Completed | RCT | India | COVID-19 with vitamin D deficiency | Vitamin D | 60.000 IU once | 60000 IU followed by 60000 IU daily or weekly depending on levels | daily | 7 days | Yes | Yes | 40 |  |
| NCT04641195 | Recruting | RCT | India | COVID-19 | Vitamin D | Placebo (and others) | 180000 IU bolus followed by 2000 IU | daily | 8 weeks |  | Yes | 700 |  |
| NCT04590274 | Withdrawn | Clinical Trial |  | COVID-19 | Vitamin D (and others) | None | 5000 IU |  |  |  |  | 0 |  |
| NCT04937556 | Recruting | RCT | Spain | COVID-19 | Vitamin D (and other supplements) | Placebo |  |  |  |  |  | 60 |  |
| NCT04525820 | Active | RCT | Switzerland | COVID-19 | Vitamin D | Placebo or bolus plus placebo | 140000 IU bolus followed by 800 IU | daily | Until discharge | Yes | Yes | 80 |  |
| NCT05002530 | Not yet recruting | RCT | China, Egypt, Saudi Arabia | COVID-19 ansomia | Vitamin D (and other supplements) | Standard Care | 600000 IU | twice | 4 weeks |  |  | 10000 |  |
| NCT04579640 | Active | RCT | United Kingdom | COVID-19 with deficiency | Vitamin D | Standard Care | High dose 3200 IU, low dose 800 IU | daily |  | Yes | Yes | 6200 |  |
| NCT04372017 | Terminated | RCT | USA | COVID-19 | Vitamin D (and HCQ) | None | 1600 IU bolus followed by 800 IU | daily | 5 days |  |  |  |  |
| NCT04489628 | Withdrawn | RCT | USA | COVID-19 | Vitamin D | Placebo | 50000 IU |  |  |  |  | 0 |  |
| NCT04609423 | Recruting | RCT | Norway | COVID-19 and other infections | Cod liver oil | Placebo | 400 IU | daily | 6 months | Yes | Yes | 80000 |  |
| NCT04335084 | Recruting | RCT | USA | COVID-19 | Vitamin D (and HCQ, vitamin C, zinc) | Placebo |  |  | 12 weeks |  |  | 600 |  |
| NCT04482686 | Active | RCT | USA | COVID-19 | Vitamin D (and others) | Placebo |  |  | 10 days |  |  | 31 |  |
| NCT05077813 | Not yet recruting | RCT | Egypt, Saudi Arabia | COVID-19 and Tuberculosis | Vitamin D (and others) | Standard Care | 600000 IU | twice | 4 weeks | Yes | Yes | 250 |  |
| NCT04810949 | Terminated | RCT | Mexico | COVID-19 and other infections | Vitamin D | Sun exposure and nutrition | 4000 IU | 13 times a month |  | Yes |  | 41 |  |
| NCT04845971 | Completed | Clinical Trial | Italy | COVID-19 | Vitamin D (and others) | None | 10000 IU | daily |  | Yes | Yes | 97 |  |
| NCT04476745 | Enrolling | RCT | Jordan | Cytokine storm | Vitamin D | No treatment | 50000 IU | weekly | 8 weeks | Yes | Yes | 100 |  |
| NCT04621058 | Recruting | RCT | Spain | COVID-19 | Vitamin D | Placebo | 267 IU or double dose |  |  | Yes | Yes | 108 |  |
| NCT04751669 | Not yet recruting | RCT | Spain | COVID-19 | Vitamin D (and other supplements) | Placebo | 400 IU |  |  | Yes | Yes | 300 |  |
| NCT04334512 | Recruting | RCT | USA | COVID-19 | Vitamin D (and others) | Placebo |  |  |  |  |  | 600 |  |
| NCT03188796 | Recruting | RCT | Austria | COVID-19 | Vitamin D | Placebo | 540000 IU bolus followed by 4000 IU | daily | 90 days | Yes |  | 2400 |  |
| NCT04756856 | Recruting | Clinical Trial | Italy | COVID-19 and Sarcopenia | Vitamin D (and other supplements) | None | 16000 IU | daily | 12 weeks | Yes | Yes | 50 |  |
| NCT04386850 | Recruting | RCT | Iran | COVID-19 (treatment and prevention) | Vitamin D | Placebo | 1000 IU | daily | 2 months | Yes | Yes | 1500 | Criteria fulfilled |
| NCT04596657 | Active | RCT | USA | COVID-19 | Vitamin D | No treatment | 5000 IU | daily |  |  |  | 876 |  |

(4)

Abbreviations: DM: diabetes mellitus; IU: international units; NCT: ClinicalTrials.gov identifier; RCT: randomized controlled trial

**Supplementary Table 5.2** Analysis of ongoing trials registered on ClinicalTrials.gov.

| **Trials…** | **Number of trials** | **Percentages** |
| --- | --- | --- |
| **… only investigating COVID-19 and SARS-CoV-2 infections** | 45/50 | 90 % |
| **… only administrating vitamin D** | 35/50 | 70 % |
| **… with control group** | 45/50 | 90 % |
| **… with placebo control** | 26/50 | 52 % |
| **… with dose ≥ 1000 IU vitamin D** | 42/50 | 84 % |
| **… with dose ≥ 10000 IU vitamin D** | 29/50 | 58 % |
| **… with dose ≥ 100000 IU vitamin D** | 13/50 | 16 % |
| **… administrating vitamin D daily** | 23/50 | 26 % |
| **… administrating vitamin D daily for ≥ 4 weeks or 1 month** | 15/50 | 30 % |
| **… administrating vitamin D daily for ≥ 12 weeks or 3 months** | 7/50 | 14 % |
| **… assessing vitamin D serum levels at baseline** | 25/50 | 50 % |
| **… adjusting vitamin D serum levels at baseline** | 0/50 | 0 % |
| **… assessing vitamin D serum level in follow-up** | 24/50 | 48 % |
| **… with sample size ≥ 50** | 40/50 | 80 % |
| **… with sample size ≥ 100** | 30/50 | 60 % |
| **… with sample size ≥ 1000** | 7/50 | 14 % |

(4)

Abbreviations: IU: international units

**Supplementary Table 6** PRISMA Checklist

| **Section and Topic** | **Item #** | **Checklist item** | **Page where item is reported** |
| --- | --- | --- | --- |
| **TITLE** | | |  |
| Title | 1 | Identify the report as a systematic review. | 1 |
| **ABSTRACT** | | |  |
| Abstract | 2 | See the PRISMA 2020 for Abstracts checklist. | 2 |
| **INTRODUCTION** | | |  |
| Rationale | 3 | Describe the rationale for the review in the context of existing knowledge. | 3 |
| Objectives | 4 | Provide an explicit statement of the objective(s) or question(s) the review addresses. | 3 |
| **METHODS** | | |  |
| Eligibility criteria | 5 | Specify the inclusion and exclusion criteria for the review and how studies were grouped for the syntheses. | 4 |
| Information sources | 6 | Specify all databases, registers, websites, organisations, reference lists and other sources searched or consulted to identify studies. Specify the date when each source was last searched or consulted. | 3,4 |
| Search strategy | 7 | Present the full search strategies for all databases, registers and websites, including any filters and limits used. | 3,4 |
| Selection process | 8 | Specify the methods used to decide whether a study met the inclusion criteria of the review, including how many reviewers screened each record and each report retrieved, whether they worked independently, and if applicable, details of automation tools used in the process. | 3,4 |
| Data collection process | 9 | Specify the methods used to collect data from reports, including how many reviewers collected data from each report, whether they worked independently, any processes for obtaining or confirming data from study investigators, and if applicable, details of automation tools used in the process. | 4 |
| Data items | 10a | List and define all outcomes for which data were sought. Specify whether all results that were compatible with each outcome domain in each study were sought (e.g. for all measures, time points, analyses), and if not, the methods used to decide which results to collect. | 4 |
|  | 10b | List and define all other variables for which data were sought (e.g. participant and intervention characteristics, funding sources). Describe any assumptions made about any missing or unclear information. | 4 |
| Study risk of bias assessment | 11 | Specify the methods used to assess risk of bias in the included studies, including details of the tool(s) used, how many reviewers assessed each study and whether they worked independently, and if applicable, details of automation tools used in the process. | 4 |
| Effect measures | 12 | Specify for each outcome the effect measure(s) (e.g. risk ratio, mean difference) used in the synthesis or presentation of results. | 4 |
| Synthesis methods | 13a | Describe the processes used to decide which studies were eligible for each synthesis (e.g. tabulating the study intervention characteristics and comparing against the planned groups for each synthesis (item #5)). | 4 |
|  | 13b | Describe any methods required to prepare the data for presentation or synthesis, such as handling of missing summary statistics, or data conversions. | 4 |
|  | 13c | Describe any methods used to tabulate or visually display results of individual studies and syntheses. | 4 |
|  | 13d | Describe any methods used to synthesize results and provide a rationale for the choice(s). If meta-analysis was performed, describe the model(s), method(s) to identify the presence and extent of statistical heterogeneity, and software package(s) used. | 4 |
|  | 13e | Describe any methods used to explore possible causes of heterogeneity among study results (e.g. subgroup analysis, meta-regression). | 4 |
|  | 13f | Describe any sensitivity analyses conducted to assess robustness of the synthesized results. | 4 |
| Reporting bias assessment | 14 | Describe any methods used to assess risk of bias due to missing results in a synthesis (arising from reporting biases). | 4 |
| Certainty assessment | 15 | Describe any methods used to assess certainty (or confidence) in the body of evidence for an outcome. | 4 |
| **RESULTS** | | |  |
| Study selection | 16a | Describe the results of the search and selection process, from the number of records identified in the search to the number of studies included in the review, ideally using a flow diagram. | 4,5 |
|  | 16b | Cite studies that might appear to meet the inclusion criteria, but which were excluded, and explain why they were excluded. | 4,5 |
| Study characteristics | 17 | Cite each included study and present its characteristics. | 5 |
| Risk of bias in studies | 18 | Present assessments of risk of bias for each included study. | 5 |
| Results of individual studies | 19 | For all outcomes, present, for each study: (a) summary statistics for each group (where appropriate) and (b) an effect estimate and its precision (e.g. confidence/credible interval), ideally using structured tables or plots. | 5,6 |
| Results of syntheses | 20a | For each synthesis, briefly summarise the characteristics and risk of bias among contributing studies. | 5,6 |
|  | 20b | Present results of all statistical syntheses conducted. If meta-analysis was done, present for each the summary estimate and its precision (e.g. confidence/credible interval) and measures of statistical heterogeneity. If comparing groups, describe the direction of the effect. | 5,6 |
|  | 20c | Present results of all investigations of possible causes of heterogeneity among study results. | 5,6 |
|  | 20d | Present results of all sensitivity analyses conducted to assess the robustness of the synthesized results. | 5,6 |
| Reporting biases | 21 | Present assessments of risk of bias due to missing results (arising from reporting biases) for each synthesis assessed. | 6 |
| Certainty of evidence | 22 | Present assessments of certainty (or confidence) in the body of evidence for each outcome assessed. | 6 |
| **DISCUSSION** | | |  |
| Discussion | 23a | Provide a general interpretation of the results in the context of other evidence. | 6,7 |
|  | 23b | Discuss any limitations of the evidence included in the review. | 7 |
|  | 23c | Discuss any limitations of the review processes used. | 7 |
|  | 23d | Discuss implications of the results for practice, policy, and future research. | 7,8 |
| **OTHER INFORMATION** | | |  |
| Registration and protocol | 24a | Provide registration information for the review, including register name and registration number, or state that the review was not registered. | 3 |
|  | 24b | Indicate where the review protocol can be accessed, or state that a protocol was not prepared. | 3 |
|  | 24c | Describe and explain any amendments to information provided at registration or in the protocol. | 3 |
| Support | 25 | Describe sources of financial or non-financial support for the review, and the role of the funders or sponsors in the review. | 12 |
| Competing interests | 26 | Declare any competing interests of review authors. | 12 |
| Availability of data, code and other materials | 27 | Report which of the following are publicly available and where they can be found: template data collection forms; data extracted from included studies; data used for all analyses; analytic code; any other materials used in the review. | 12 |

*From:*  Page MJ, McKenzie JE, Bossuyt PM, Boutron I, Hoffmann TC, Mulrow CD, et al. The PRISMA 2020 statement: an updated guideline for reporting systematic reviews. BMJ 2021;372:n71. doi: 10.1136/bmj.n71

For more information, visit: http://www.prisma-statement.org/

*
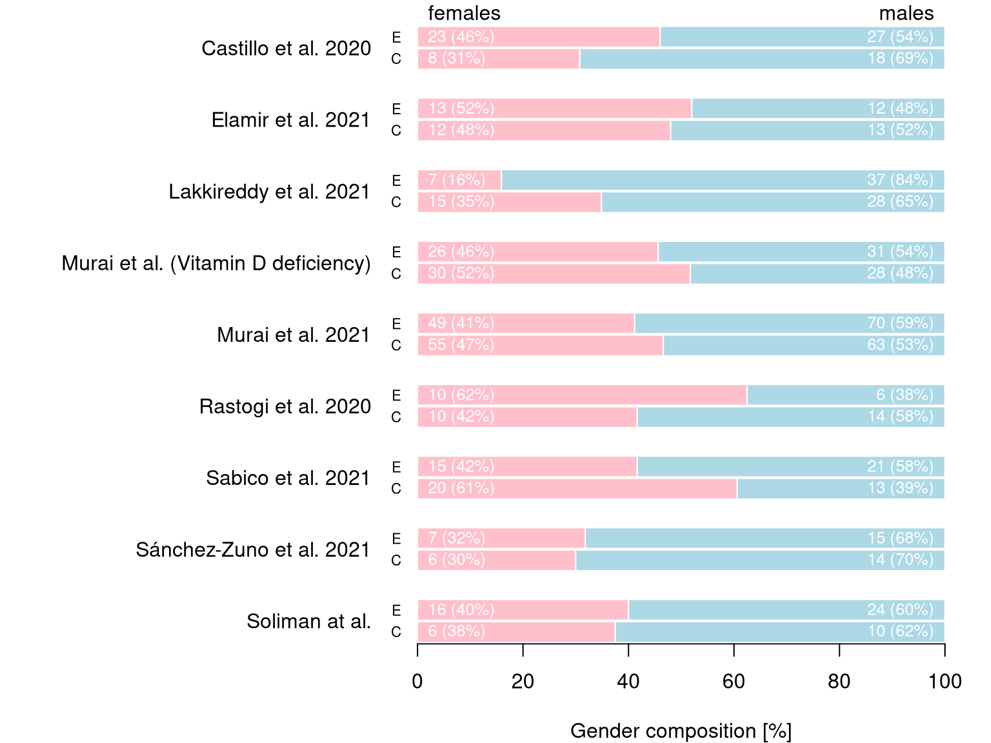
*

**Supplementary Figure 1** Proportion of gender per study, with an over proportion of 43.15% being female

**Supplementary Figure 2** Overall results of the risk of bias assessment


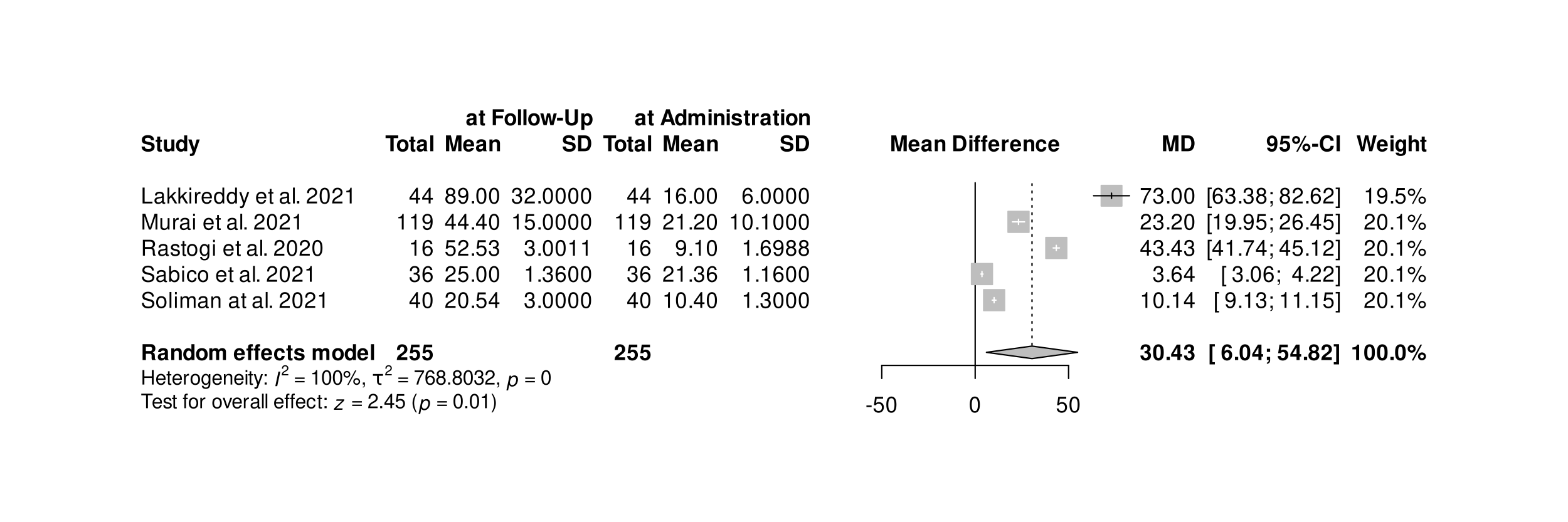


**Supplementary Figure 3** Forest plot showing the increase of vitamin D serum levels in patients, after receiving the intervention

Abbreviations: CI: confidence interval; MD: mean difference; SD: standard deviation

*
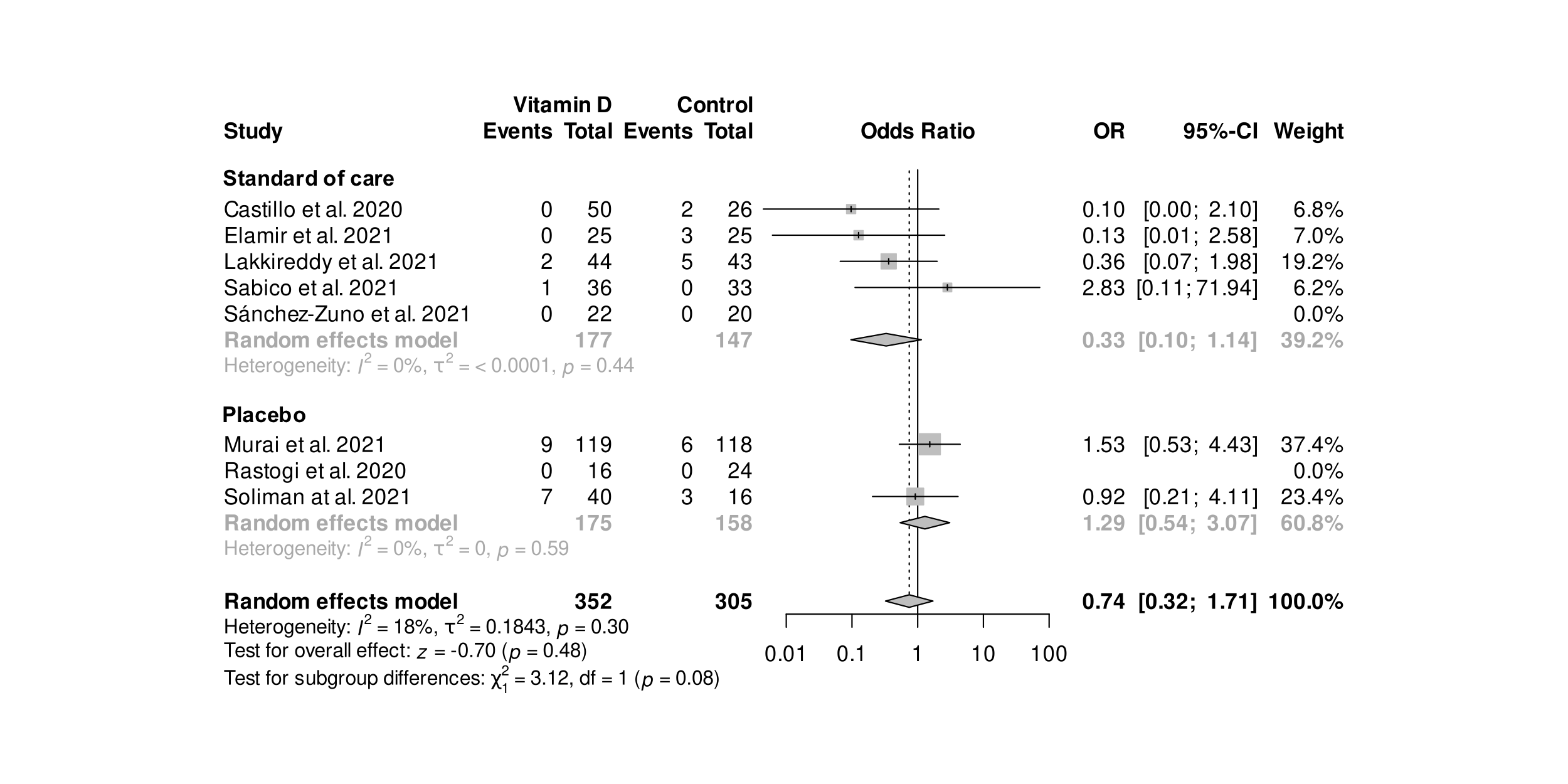
*

**Supplementary Figure 4** Forest plot with subgroup analysis showing the effect of vitamin D supplementation compared to placebo and standard of care

Abbreviations: CI: confidence interval; OR: odds ratio


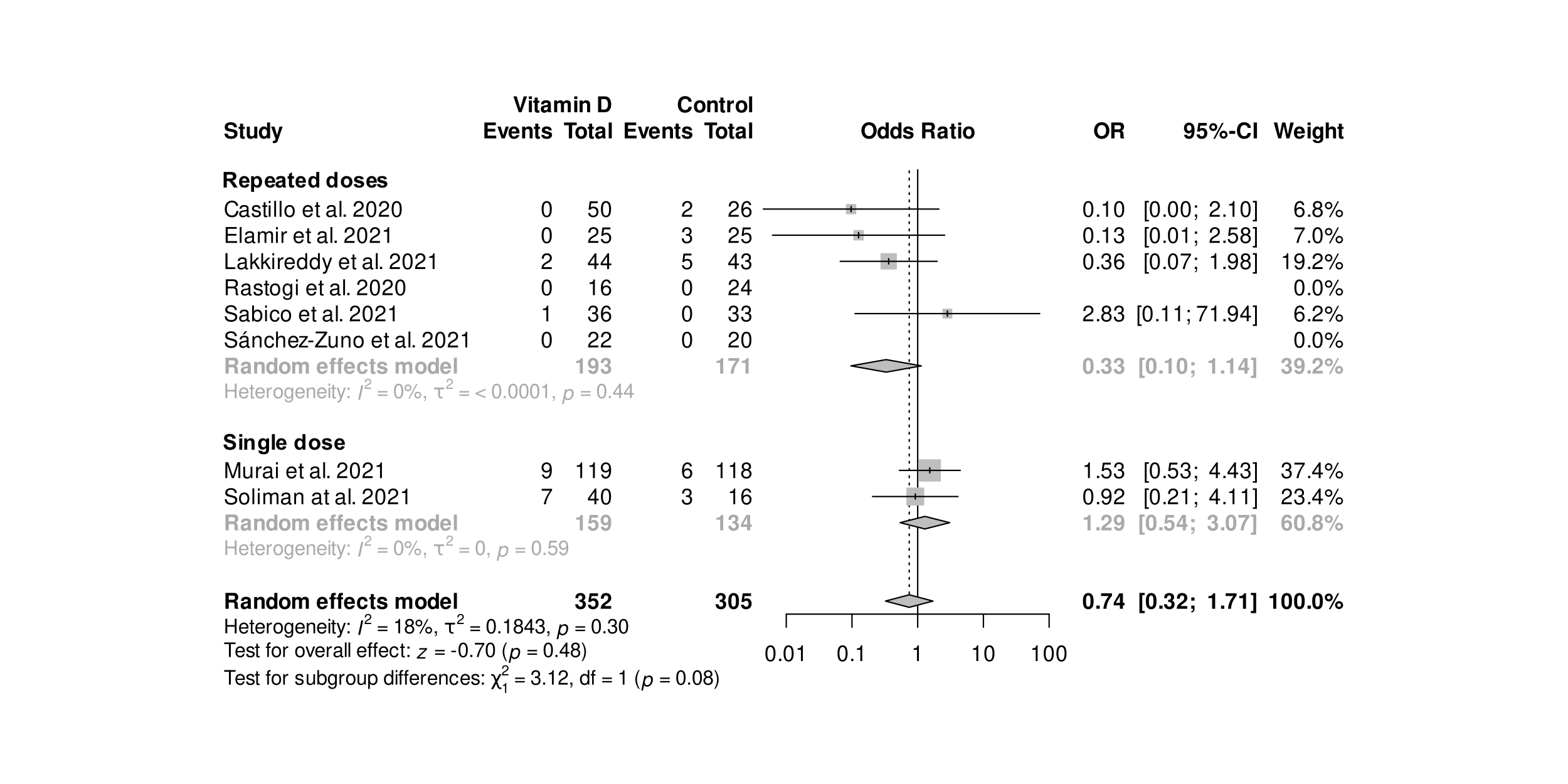


**Supplementary Figure 5** Forest plot with subgroup analysis showing the effect of single or repeated dosages of vitamin D on mortality

Abbreviations: CI: confidence interval; OR: odds ratio


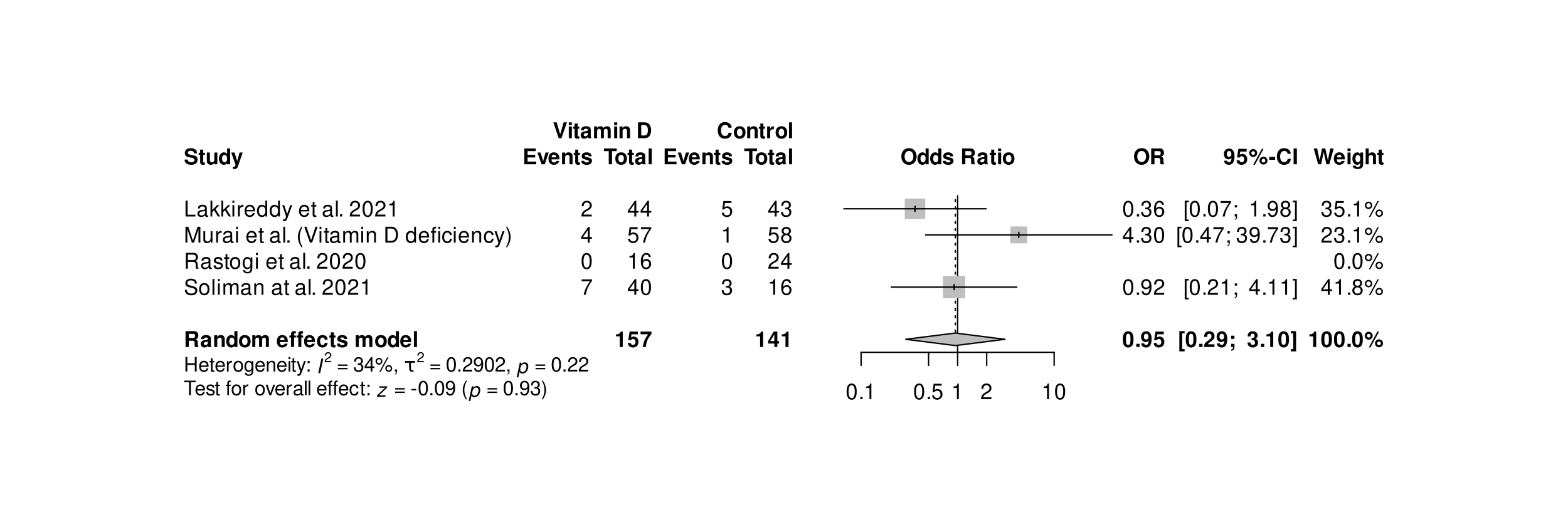


**Supplementary Figure 6** Forest plot with subgroup analysis showing the effect of vitamin D supplementation on mortality in patients with confirmed vitamin D deficiency (as defined by study investigators)

Abbreviations: CI: confidence interval; OR: odds ratio

*
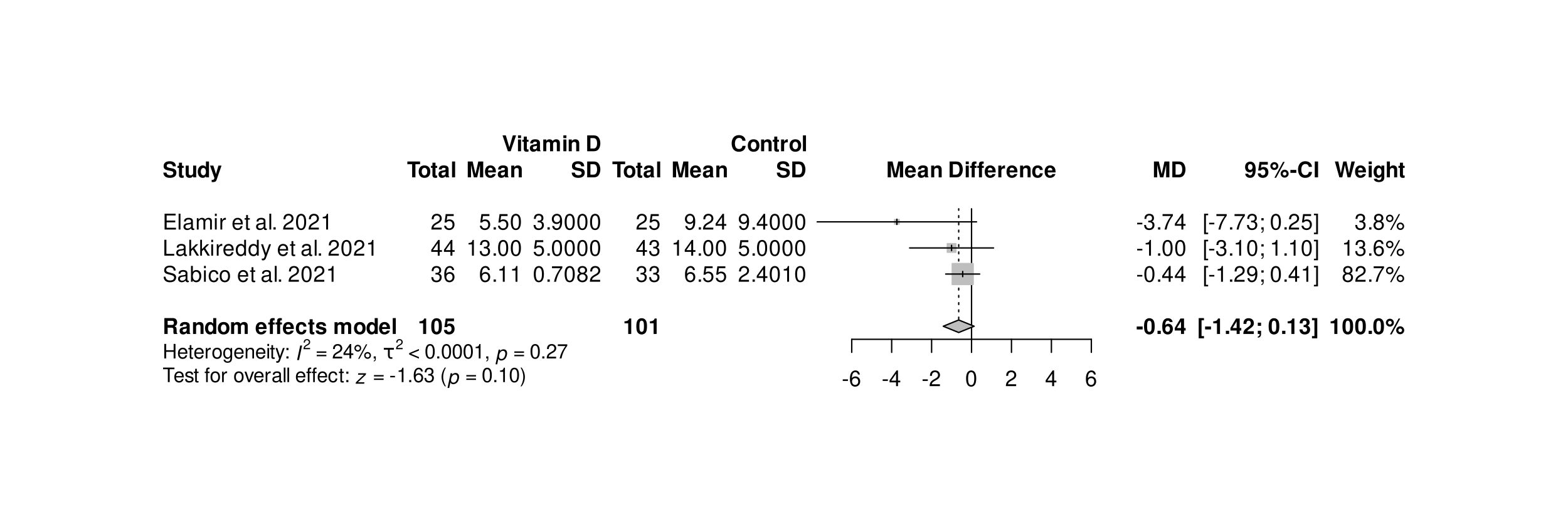
***Supplementary Figure 7** Forest plot with subgroup analysis showing the effect of repeated vitamin D supplementation on the length of hospitalization

Abbreviations: CI: confidence interval; OR: odds ratio

*
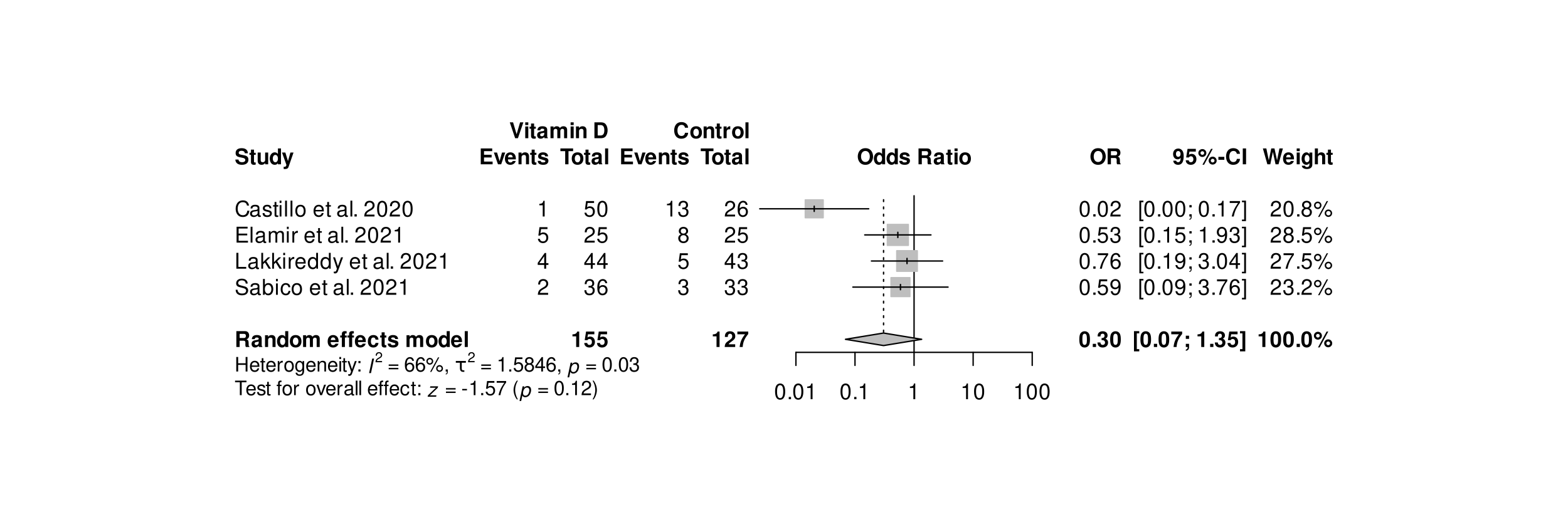
***Supplementary Figure 8** Forest plot with subgroup analysis showing the effect of repeated vitamin D supplementation on the need for ICU admission

Abbreviations: CI: confidence interval; OR: odds ratio

*
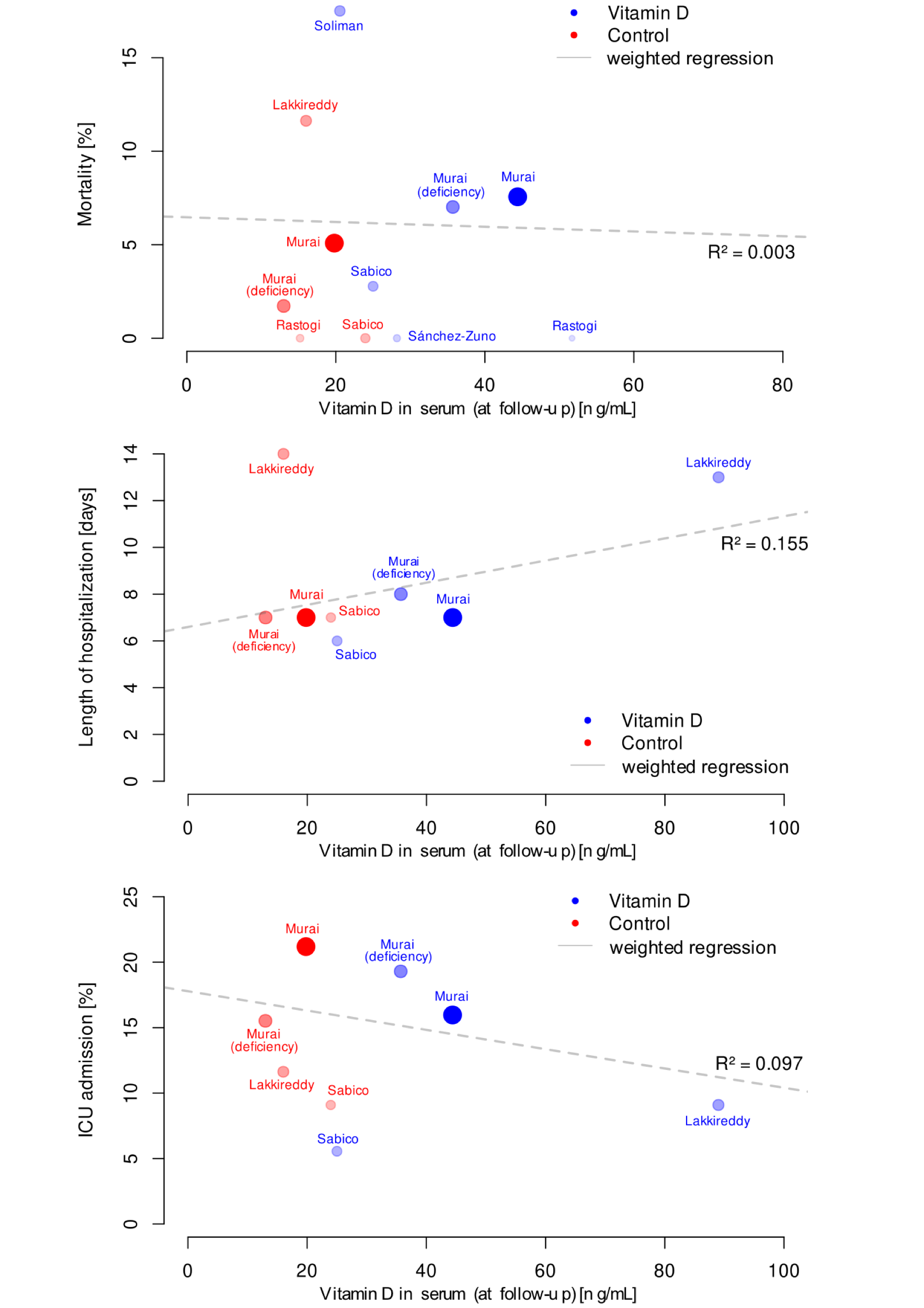
*

**Supplementary Figure 9** Correlation of vitamin D serum levels and various outcomes weighted by study size: **A** Mortality; **B** Length of hospitalization; **C** ICU admission


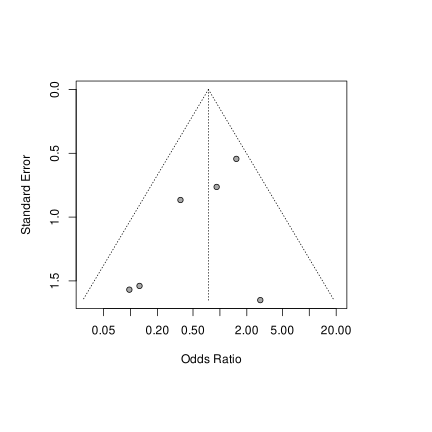


**Supplementary Figure 10** Funnel plot for mortality assessing risk of publication bias

**References**

1. WHO COVID-19: Case Definitions. World Health Organization: WHO; 2020 16.12.2020. Report No.: WHO/2019-nCoV/Surveillance_Case_Definition/2020.2.

2. Health NIo. COVID-19 Treatment Guidelines Panel. Coronavirus Disease 2019 (COVID-19).

3. Health NIo. VItamin D 2021 [updated 17.08.2021. Available from: <https://ods.od.nih.gov/factsheets/VitaminD-HealthProfessional/>.

4. : NIH: U.S. National Library of Medicine; [Available from: <https://clinicaltrials.gov>.
